# Evaluation of genomic high-throughput sequencing data generated on Illumina HiSeq and Genome Analyzer systems

**DOI:** 10.1186/gb-2011-12-11-r112

**Published:** 2011-11-08

**Authors:** André E Minoche, Juliane C Dohm, Heinz Himmelbauer

**Affiliations:** 1 Max Planck Institute for Molecular Genetics, Ihnestr. 63-73, 14195 Berlin, Germany; 2 Centre for Genomic Regulation (CRG) and UPF, C. Dr. Aiguader 88, 08003 Barcelona, Spain

## Abstract

**Background:**

The generation and analysis of high-throughput sequencing data are becoming a major component of many studies in molecular biology and medical research. Illumina's Genome Analyzer (GA) and HiSeq instruments are currently the most widely used sequencing devices. Here, we comprehensively evaluate properties of genomic HiSeq and GAIIx data derived from two plant genomes and one virus, with read lengths of 95 to 150 bases.

**Results:**

We provide quantifications and evidence for GC bias, error rates, error sequence context, effects of quality filtering, and the reliability of quality values. By combining different filtering criteria we reduced error rates 7-fold at the expense of discarding 12.5% of alignable bases. While overall error rates are low in HiSeq data we observed regions of accumulated wrong base calls. Only 3% of all error positions accounted for 24.7% of all substitution errors. Analyzing the forward and reverse strands separately revealed error rates of up to 18.7%. Insertions and deletions occurred at very low rates on average but increased to up to 2% in homopolymers. A positive correlation between read coverage and GC content was found depending on the GC content range.

**Conclusions:**

The errors and biases we report have implications for the use and the interpretation of Illumina sequencing data. GAIIx and HiSeq data sets show slightly different error profiles. Quality filtering is essential to minimize downstream analysis artifacts. Supporting previous recommendations, the strand-specificity provides a criterion to distinguish sequencing errors from low abundance polymorphisms.

## Background

Next generation sequencing (NGS) is revolutionizing molecular biology research with a wide and rapidly growing range of applications. These applications include *de novo *genome sequencing, re-sequencing, detection and profiling of coding and non-coding transcripts, identification of sequence variants, epigenetic profiling, and interaction mapping. Compared with microarrays, previously used for many of these applications, NGS offers a higher dynamic range, enabling the detection of rare transcripts and splice variants in the transcriptome as well as rare genomic polymorphisms - for example, somatic mutations present within cancer samples. The challenge remains to distinguish sequence variation from sequencing errors, and a thorough characterization of NGS data is required in order to detect method-inherent errors and biases. Systematic errors are platform-dependent. In the context of this work, we focus on Illumina data. According to market share analysis, almost two thirds of all NGS instruments presently in operation have been manufactured by Illumina [1].

Existing studies about Illumina data evaluation have revealed several biases, that is, a non-random distribution of the reads in the sequenced sample over the reference (reported for the Genome Analyzer (GA) I [2-5]) and a non-random distribution of errors (GAIIx [6]). Preferences of certain substitution errors and sequence context have been observed. For instance, wrong base calls are frequently preceded by base G [2] and frequencies of base substitutions vary by 10- to 11-fold, with A to C conversions being the most frequent error [2,7]. Such errors might have profound implications on the interpretation of results: a non-random read distribution can bias profiling of transcripts and hamper the detection of sequence polymorphisms in regions of low sequence coverage. Errors in the reads can result in false positive variant calls or wrong consensus sequences.

The Illumina sequencing technology has been under constant development, relating to instrumentation, signal processing software, and sequencing chemistry, towards the production of more data and longer sequencing reads. The HiSeq2000 became commercially available in the second quarter of 2010 and uses sequencing-by-synthesis (SBS) chemistry similar to the Illumina GA series but at a two- to five-fold increased rate of data acquisition. A HiSeq flow cell can be imaged on both the top and bottom surface. To increase the HiSeq data collection rate, imaging is performed in a line scanning mode, in contrast to the area imaging in the GA. Instead of using only one camera, the HiSeq operates with a four camera system that detects the intensities of all four bases simultaneously. The Hiseq currently runs with lower cluster densities than the GA and with a maximal read length of 100 nucleotides for single reads or 2 × 100 nucleotides in paired-end mode.

Every development of a system can shift error profiles and can reveal new types of errors. Here, we evaluate Illumina sequencing data generated on the latest systems, the GAIIx and HiSeq2000, using current sequencing chemistry and up-to-date base-calling software. We focus on errors and biases that have an impact on common sequencing applications and we provide suggestions on how to trim and filter the reads in order to substantially reduce error rates. Since high quality reference sequences are not always available in a sequencing project, we first report properties of the unprocessed raw reads. Then we assess the error rates and biases after mapping against high quality reference sequences derived from two plants (*Beta vulgaris *and *Arabidopsis thaliana*) and the bacterial virus PhiX174.

## Results

We generated genomic paired-end reads of 2 × 95 nucleotides and 2 × 100 nucleotides on an Illumina HiSeq2000 sequencing machine and of 2 × 150 nucleotides on an Illumina GAIIx instrument (Table 1). Three HiSeq flowcell lanes of 2 × 95-nucleotide reads resulted in 246 million read pairs corresponding to 46.8 billion bases of sequence data. These data were a mix of genomic reads of *B. vulgaris *(Bv, 99%) and the bacteriophage PhiX174 (PhiX, 1%) spiked in as standard quality control. One HiSeq flowcell lane of 2 × 100-nucleotide read pairs containing 99% genomic DNA of *A. thaliana *(At) and 1% PhiX resulted in 71 million read pairs corresponding to 14.3 billion sequenced bases. One lane containing PhiX only was sequenced on a GAIIx and yielded 9 million read pairs of length 2 × 150 nucleotides (2.7 billion bases).

**Table 1:** Properties of the sequence data

Species	Bv + PhiX	At + PhiX	PhiX
Platform	HiSeq	HiSeq	GAIIx
Read length	95	100	150
Number of lanes	3	1	1
Number of sequenced pairs	246,159,940	71,393,237	9,046,254
Number of sequenced bases	46,770,388,600	14,278,647,400	2,713,876,200
Fraction of uncalled bases	1.52%	1.21%	13.77%
Fraction of uncalled bases - read 1	1.45%	1.07%	12.66%
Fraction of uncalled bases - read 2	1.58%	1.34%	14.87%
Fraction of reads with at least one uncalled base	2.46%	2.26%	15.57%
Fraction of entirely uncalled reads	0.56%	0.50%	7.16%
Fraction of bases in B-tails	11.01%	16.56%	25.78%
Fraction of uncalled bases in B-tails	1.49%	1.18%	13.75%
Fraction of bases in B-tails - read 1	11.02%	14.45%	24.82%
Fraction of bases in B-tails - read 2	11.00%	18.67%	26.74%
Average length of B-tails	10.5 (9.9)^a^	16.6 (16.1)^a^	38.7 (27.9)^a^
Fraction of reads with B-tail	26.15%	39.44%	67.87%
Fraction of reads containing at least one uncalled base in B-tail	2.19%	1.98%	14.99%
Fraction of both reads with B-tail	14.70%	24.50%	53.10%
Average Q-score	31.81	30.23	27.17
Average Q-score - read 1	31.83	31.10	27.62
Average Q-score - read 2	31.80	29.37	26.73
Q ≥ 30 bases	37.27 Gbp (79.68%)	10.56 Gbp (73.99%)	1.74 Gbp (64.29%)
Q ≥ 30 bases - read 1	18.70 Gbp (39.98%)	5.49 Gbp (38.42%)	0.90 Gbp (33.01%)
Q ≥ 30 bases - read 2	18.57 Gbp (39.71%)	5.08 Gbp (35.57%)	0.85 Gbp (31.28%)

**^a^**Values in parentheses are those without reads entirely composed of uncalled bases. At, *Arabidopsis thaliana*; Bv, *Beta vulgaris*; PhiX, bacteriophage PhiX174.

### Properties of raw reads and filtering criteria

As a first quality evaluation we analyzed the raw read sequences and their corresponding quality values assigned by the base-calling software. The Illumina base-calling software calculates a quality score for each base reflecting the probability that the called base is wrong. The calculation takes into account the ambiguity of the signal for the respective base as well as the quality of neighboring bases and the quality of the entire read. The quality score Q is defined by Q = -10 log_10_(P); for example, Q = 30 corresponds to the probability *P *= 0.001 that a base has been called incorrectly. The highest possible value for Q assigned by the base-calling software is 40, corresponding to *P *= 0.0001.

In the samples sequenced on the HiSeq, 80% (Bv + PhiX, read length 95 nucleotides) and 74% (At + Phix, 100 nucleotides) of all bases had quality scores of at least 30, whereas for the PhiX data (150 nucleotides) sequenced on the GAIIx this fraction was 64%. The average quality score was Q = 31.8 (Bv + PhiX) and Q = 30.2 (At + PhiX) for the HiSeq data and Q = 27.2 for the GAIIx data. For both platforms the first read of a read-pair had slightly better average quality scores than the second read. The difference of Q between both reads was in the range of 0.3 (HiSeq) and 1.7 (GAIIx), respectively.

Uncalled bases are represented by a 'dot' in the sequence and by a 'B' in the quality string (corresponding to a quality score of Q = 2; the quality values are represented by ASCII characters). In the entire HiSeq data set 1.4% of all bases were uncalled, affecting 2.4% of all reads, and 0.5% of all reads were entirely composed of uncalled bases. In the GAIIx data set we found 14% of all bases to be uncalled, affecting 16% of all reads, and 7% of all reads were entirely uncalled (Table 1).

The quality of the 3' end of a sequencing read can be low for reasons such as phasing artifacts. If most bases at the 3' end of a read have quality values of Q ≤ 15, the base-calling software considers the whole segment as unreliable and assigns values of Q = 2 to the bases of this segment (represented by a 'B' in the quality string, just like uncalled bases). Illumina recommends excluding this portion of the read in further analysis (CASAVA1.7 User Guide). In the following we use the term 'B-tail' for consecutive Bs at the 3' end of a read, including unreliably called bases as well as uncalled bases. The most extreme cases - that is, reads entirely composed of Bs or reads containing only one B at the 3' end - are also considered as B-tailed reads. The fraction of bases lying within B-tails was 13.8% in the HiSeq data and 25.8% in the GAIIx data. Among these B-tail bases, 10.3% (HiSeq) and 53.3% (GAIIx) were uncalled. The B-tail length distribution shows a slight increase towards short B-tails and a sharp increase towards reads entirely composed of B. The predominant size is the full-length B-tail even after the removal of reads entirely composed of uncalled bases (Figure S3 in Additional file 1). In both HiSeq data sets, on average, 32.8% of all reads we studied had B-tails, and 19.6% of all read pairs had a B-tail in both reads. In the GAIIx data 67.9% of all reads had a B-tail, and 53% of all read pairs had a B-tail in both reads. Excluding the reads entirely composed of uncalled bases, the average read length after B-tail trimming was reduced to 122 bases in GAIIx reads (original read length 150 nucleotides), to 85 bases for reads from the HiSeq Bv + PhiX sample (original read length 95 nucleotides) and to 74 bases for the HiSeq At + PhiX reads (original read length 100 nucleotides).

Removing B-tails has a strong effect on the expected error rate (determined by the average of the error probability of each base according to Illumina quality values). In HiSeq Bv + PhiX data the removal of B-tails decreased the expected error rate from 7.09% to 0.16% and reduced the data output by 11%. In the GAIIx data the expected error rate decreased from 16.43% to 0.23%, reducing the data amount by 25.8%. Apart from B-tail removal, further filters can be applied based on Illumina's quality measurement. We tested several filtering criteria separately and in combination and recorded the resulting expected error rates (Table 2). The Illumina software provides a read quality rating by introducing the chastity filter. The chastity is determined from the ratio of the signal intensities of the four possible bases in each sequencing cycle. Reads do not pass the chastity filter if they underrun a certain chastity cutoff within the first 25 cycles (see Materials and methods for details). The lowest expected error rate was obtained for the following combination of filtering criteria: B-tail trimming, passed chastity filter, removal of reads containing uncalled bases, keeping reads only if at least two-thirds of the bases of the first half of the read had quality values of Q ≥ 30.

**Table 2:** Expected error rates after filtering

	**Expected error rate**^**a **^**(percentage of bases discarded)**		
Filter	Bv + PhiX	At + PhiX	PhiX-GAIIx
No filter	7.093 (0.0%)	10.619 (0.0%)	16.434 (0.0%)
ChF	2.583 (10.2%)	4.819 (12.7%)	7.360 (17.8%)
B-tail	0.163 (11.0%)	0.205 (16.6%)	0.229 (25.8%)
N	5.943 (2.5%)	9.688 (2.3%)	8.601 (15.6%)
C33	1.521 (14.3%)	2.907 (17.7%)	5.207 (21.7%)
A30	1.802 (12.7%)	3.586 (15.5%)	5.457 (21.3%)
B-tail + N	0.141 (11.4%)	0.187 (17.0%)	0.206 (26.8%)
B-tail + ChF	0.118 (13.8%)	0.161 (19.2%)	0.204 (27.2%)
B-tail + ChF + C33	0.083 (16.9%)	0.127 (22.1%)	0.174 (28.3%)
B-tail + ChF + A30	0.093 (15.8%)	0.139 (21.0%)	0.176 (28.2%)
B-tail + ChF + N + C33	0.077 (17.1%)	0.125 (22.2%)	0.168 (28.9%)
B-tail + ChF + N + A30	0.085 (16.0%)	0.136 (21.1%)	0.171 (28.6%)

**^a^**Expected error rate: average error probability of each base assigned by Illumina as quality scores. No filter, raw data prior to filtering; ChF, Illumina chastity filter; B-tail, B-tail trimming; N, removal of reads with at least one uncalled base; C33, removal of reads having less than two-thirds of bases with Q ≥ 30 within the first half of the read; A30, removal of reads that have an average Q-score < 30 in the first 30% of the read. Note that some quality filters discard partially the same reads (for instance, the chastity filter and the removal of reads containing at least one uncalled base).

The GC content (%GC) of the unfiltered HiSeq reads was higher than expected: 40% for Bv + PhiX data and 45.5% for At + PhiX. The *B. vulgaris *reference sequence has a %GC of 35% [8] and that of the *A. thaliana *genome is 36% (calculated from TAIR10 [9]). The fraction of PhiX reads (44.7% GC) accounts for only 1 to 2% of the data. For the PhiX sample sequenced on the GAIIx the %GC of 45.7% is much closer to the expected value of 44.7%.

### Mapping of raw reads against reference sequences

We evaluated the actual quality of the sequencing reads by mapping the reads against high-quality reference sequences. We used the 5-kbp bacteriophage PhiX sequence, the 110-kbp insert sequence of a *B. vulgaris *BAC clone, and the 30 Mbp chromosome 1 of *A. thaliana *as references (see Materials and methods). The small and gene-dense PhiX genome is commonly used in Illumina sequencing as quality control. Sugar beet has a highly repetitive genome, and from *Arabidopsis *we used the large sequence of an entire chromosome in order to include references of different lengths and properties in our study.

We mapped the whole data set against the PhiX reference genome (5,386 bp) and kept all read pairs that had passed the Illumina chastity filter, did not contain adapter sequence, and were matching the genome uniquely with correct read orientation and expected mapping distance. This resulted in 4,302,400 and 887,009 PhiX read pairs sequenced on the HiSeq (2 × 95 nucleotides together with sugar beet or 2 × 100 nucleotides together with *Arabidposis*, respectively) and 6,405,298 PhiX read pairs sequenced on the GAIIx (2 × 150 nucleotides). To distinguish these three PhiX data sets in the following analysis, we use the terms PhiX-95nt, PhiX-100nt, and PhiX-GAIIx.

The sugar beet sample is derived from a whole genome shotgun library that was sequenced in three HiSeq lanes. The reference is the BAC insert ZR-47B15 (109,563 bp), here called 'ZR', sequenced to finished quality [8] and previously used in a study on the quality of Illumina reads produced on the GA I sequencing instrument [2]. We implemented filtering steps for sugar beet reads in order to exclude reads that mapped to ZR but originated from a different region of the genome (see Materials and methods). Such wrongly assigned reads could lead to erroneous conclusions on read coverage and read error rates - for instance, in the case of divergent repetitive regions. We obtained 53,101 reads covering ZR (26,495 pairs, 111 singletons). This read data set is referred to as Bv-95nt in the following.

The *Arabidopsis *whole genome shotgun sequencing data were mapped against the entire *Arabidopsis *genome sequence. Pairs were kept if they had passed the Illumina chastity filter and matched chromosome 1 uniquely with correct read orientation and expected mapping distance, resulting in 5,815,990 pairs (referred to as the At-100nt data set).

### Read distribution over the reference sequence

**Figure 1:**
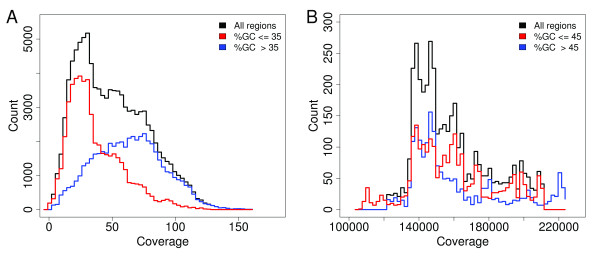
**Distribution of read coverage depth for **(a) **Bv-95nt reads and **(b) **Phix-95nt reads**. Read coverage was computed per base. In three separate calculations we considered all positions (black), positions in regions below (red) and positions in regions above (blue) the average GC content (%GC) of the reference. The regional %GC was determined based on a window of 250 bases upstream and 250 bases downstream of each position. In contrast to PhiX (b) the coverage variation in the sugar beet sample (a) is related to the %GC.

For most sequencing applications it is desired to get an even distribution of reads along the reference. Improvements in the cluster generation and sequencing chemistry may have led to a reduction of the previously observed biases [2,3]. However, we still observe high coverage variation over the ZR reference, and even in the deeply covered PhiX genome we observe variation by a factor of 2. In the sugar beet sample, the per-base coverage of ZR ranged from 0- to 159-fold, with an average of 49-fold (Figure 1a). The PhiX genome was covered, on average, 159,300-fold (range 106,500- to 224,000-fold) by PhiX-95nt data (Figure 1b), 34,710-fold (range 23,280- to 49,560-fold) by PhiX-100nt data, and 375,100-fold (range 162,100- to 508,300-fold) by PhiX-GAIIx data. Similar to previous reports, we found a positive correlation between %GC and read coverage for the two plant samples (Figures S1, S4a, b, and S6a in Additional file 1). PhiX, in contrast, did not show a significant correlation between %GC and coverage (Figures S4c-e, S5f, and S6b, c in Additional file 1). The PhiX genome differs from the plant reference sequences in its higher average %GC (PhiX, 44.7%; ZR, 34.8%; At, 35.9%) and its smaller %GC variation (1st and 99th percentiles of PhiX, 41 to 49%; of ZR, 24 to 47%; of At, 20 to 50%). Selecting ZR regions of %GC between 31% and 39% clearly showed a correlation but regions of %GC between 41% and 49% did not (data not shown). This finding suggests that the extent of the %GC-coverage correlation is dependent on the %GC range of the reference sequence.

No correlation between coverage and error rate (Figure S7a in Additional file 1) or coverage and average quality score could be detected (tested for ZR; Figure S7b in Additional file 1).

### Accumulation of reads with B-tails

**Figure 2:**
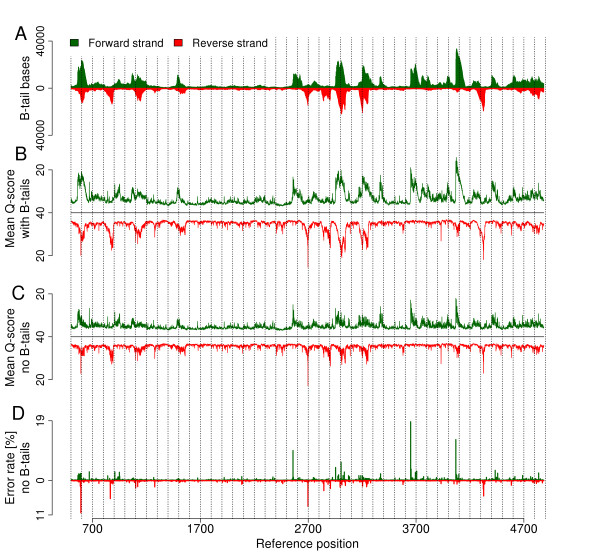
**Distribution of low quality bases along the PhiX reference genome**. Analysis was performed on reads derived from an Illumina PhiX library (PhiX-95nt data set). **(a) **Number of bases within B-tails (consecutive bases of Q-score = 2 at the 3' end of a read) per position. **(b) **Average Q-score of bases in untrimmed reads. **(c) **Average Q-score of bases in B-tail trimmed reads. **(d) **Observed per-base substitution error rate. Calculations for (a-d) were performed separately for the forward strand (green) and reverse strand (red). Low quality values accumulated in certain regions even after removal of B-tails. The peaks of observed error rates occur at positions where increased low quality counts are detected, and in most cases the peak is seen only on one strand.

Intuitively, reads with low quality 3' ends (marked with a B-tail in the quality string) are expected to occur at any position within the reference sequence. However, we noticed that B-tailed reads were not distributed randomly but accumulated at distinct locations, and in several cases the accumulation was found almost exclusively on one strand (Figure 2a; Figure S8 in Additional file 1). The average Q-scores were decreased according to where B-tails accumulated (Figure 2b), as expected, but even after B-tail trimming, regions previously spanned by B-tails still displayed lower average Q-scores in the remaining bases (Figure 2c). This observation was made in the PhiX-95nt data as well as in the Bv-95nt data and could be perfectly reproduced with the PhiX-100nt data (Figure S9a-d in Additional file 1). When comparing our PhiX-GAIIx data to the two HiSeq PhiX data sets, we observed that some of the low quality peaks were common to both sequencing platforms (Figure S9a, e in Additional file 1).

**Figure 3:**
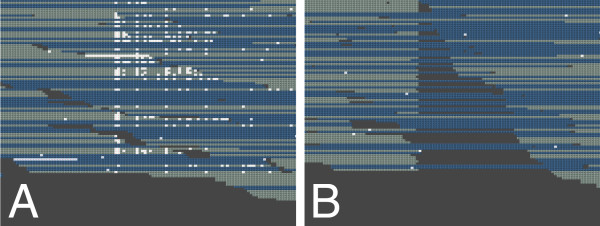
**Alignment of reads before **(a) **and after **(b) **B-tail trimming in a selected region of the ZR reference (positions 63,633 to 63,662)**. Uniquely mapped reads from the Bv-95nt data set were visualized using the Tablet browser [17]. Forward matching reads are shown in grey, reverse matching reads are shown in blue, mismatch bases are shown in white. Long white stretches are uncalled bases. Mismatches accumulated in one region, and almost all mismatches are eliminated after B-tail removal.

The example region in Figure 3 illustrates the finding of region-specific accumulation of B-tails within the ZR reference. The comparison of this region before and after B-tail trimming shows that the high number of substitution errors, densely packed within a distinct region of the reference sequence, disappears as soon as B-tails are removed (Figure 3a, b). Further analysis indicated that 95% of the B-tailed reads in this region mapped to the forward strand, suggesting a sequence context-specific accumulation of low quality reads (more precisely, of the low quality 3' parts of reads). All read pairs with a B-tail in this region had the B-tail only in one of the two reads of the pair. The accumulation of low quality bases and sequencing errors, including their directionality, was also observed by Nakamura *et al*. [6] in bacterial read data sequenced on a GAIIx, but in their study quality values were not considered as a criterion to filter out erroneous parts of the reads. They rather truncated the reads by a fixed number of bases or removed complete reads containing a certain number of mismatches. By taking off only the B-tail of a read we remove the vast majority of erroneous bases and at the same time we keep the coverage loss to a minimum. The effect of coverage decrease due to B-tail trimming is obvious for regions of B-tail accumulation. When aligning B-tail trimmed reads of Bv-95nt back to ZR, 46% of all reference bases were affected by a coverage decrease (Table 3); in some cases the coverage went down to 5% of the coverage by full reads (with B-tail). For PhiX reads, B-tail trimming reduced the coverage of each base in the genome but within a narrower range (remaining coverage 68 to 99%). However, the median coverage decreased only by 3% for both ZR and PhiX.

**Table 3:** Coverage drop after B-tail removal

Remaining per-base coverage after B-tail removal	Number of positions with this coverage in the ZR reference (Bv-95nt data)	Number of positions with this coverage in the PhiX reference (PhiX-95nt data)
90 to 100%	41,130 (40.26%)	4,016 (91.65%)
80 to 90%	4,056 (3.97%)	266 (6.07%)
60 to 80%	1,711 (1.67%)	100 (2.28%)
40 to 60%	310 (0.30%)	0 (0.00%)
20 to 40%	76 (0.07%)	0 (0.00%)
0 to 20%	29 (0.03%)	0 (0.00%)

The sequencing error analysis in the following paragraphs was performed after B-tail trimming.

### Substitution error rates and distributions

Substitution errors are far more frequent than insertions or deletions (indels) in Illumina sequencing data (Table 4). In B-tail trimmed and adapter-free PhiX-95nt reads, for instance, base substitutions account for 99.5% of all detected errors. We found 7,615 substitution errors at 6,537 different positions in the mapping result of the Bv-95nt data (6% of all ZR positions affected) and 1,792,190 substitution errors at 1,523,614 different positions in the At-100nt data (5% of all At chromosome 1 positions affected). All positions in the PhiX genome were affected by substitution errors after mapping of PhiX-GAIIx data; for HiSeq data, reduced coverage at the terminal regions of the linear PhiX reference sequence referring to a circular genome resulted in 3 bases (PhiX-95nt) and 28 bases (PhiX-100nt) remaining error-free. On average, we counted at each reference position 154 substitutions in PhiX-95nt, 37 substitutions in PhiX-100nt, and 916 substitutions in PhiX-GAIIx (reflecting the coverage difference between the three PhiX samples). This corresponds to a global average substitution error rate of 0.11% for the two HiSeq PhiX data sets and of 0.28% for the PhiX-GAIIx data set. The HiSeq read data sets from the two plant samples both had a global substitution error rate of 0.16%. Uncalled bases were not counted as sequencing errors. Within the B-tails of PhiX-95nt we find a greatly increased substitution error rate of 6.5%.

**Table 4:** Indels and substitution errors in B-tail trimmed reads

Data set	Uniquely aligned bases	Substitution errors (rate)	Indels (rate)
Bv-95nt	4,900,840	7,615 (0.16%)	84 (1.7 E-5)
PhiX-95nt	778,014,176	830,351 (0.11%)	3,789 (4.9 E-6)
At-100nt	1,111,314,053	1,792,190 (0.16%)	26,130 (2.4 E-5)
PhiX-100nt	170,078,494	203,729 (0.12%)	546 (3.2 E-6)
PhiX-GAIIx	1,760,062,929	4,936,167 (0.28%)	7,077 (4.0 E-6)

**Figure 4:**
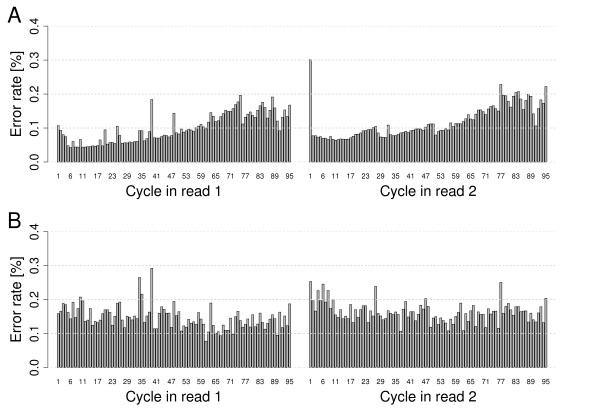
**Observed error rates of 2 × 95-nucleotide HiSeq reads by cycle (averaged across all flow cell tiles)**. Read 1 (left) and read 2 (right) were analyzed separately for PhiX-95nt data **(a) **and Bv-95nt data **(b)**. PhiX and sugar beet DNA was sequenced in the same lane, and reads were mapped against the PhiX or ZR reference sequences, respectively.

We determined the distribution of error rates within a read. In an Illumina sequencing cycle, elongation by exactly one base per molecule per cluster in the presence of all four nucleotides at the same time is taking place. We calculated the per-cycle error rate by dividing the number of base substitutions in a particular cycle by the number of all sequenced bases of that cycle. We generally observed lower per-cycle error rates in the first half of the reads, and lower error rates in read 1 compared to read 2. Per-cycle error rates ranged from 0.04 to 0.3% in PhiX-95nt reads and from 0.08 to 0.29% in Bv-95nt reads. Towards the 3' end the error rate doubles for PhiX-95nt reads (Figure 4a) and does not increase in Bv-95nt reads (Figure 4b). For the At-100nt and PhiX-100nt data sets the error rate was approximately doubled (read 1) or tripled (read 2) towards the 3' end of reads (Figure S10a, b in Additional file 1), and the Phix-GAIIx data (length 150 nucleotides) showed an error rate increase of about five- to ten-fold (Figure S10c in Additional file 1). Increased error rates up to 1.78% (about 16-fold) could be observed at 3' ends of HiSeq data if no adapter trimming was performed (Figure S10d in Additional file 1). Sequencing of library inserts smaller than the read length results in reads containing parts of the adapter. We removed reads containing adapter sequence prior to analysis (see Materials and methods).

In the sequencing data, increased error rates were observed in some sequencing cycles (Figure 4; Figure S10 in Additional file 1). It turned out that only a fraction of the reads was affected by these peaks. When inspecting their spatial placement within the flow cell we found that they concentrated in certain regions (Figure S11 and text supplement T1 in Additional file 1). The increased error rates were consistently reflected in the average quality scores of the particular cycles and regions for HiSeq reads (Figures S16 and S17 in Additional file 1) as well as GAIIx reads (data not shown). Thus, taking quality values into account should safely prevent potential interfering effects caused by these outliers during downstream analysis.

**Figure 5:**
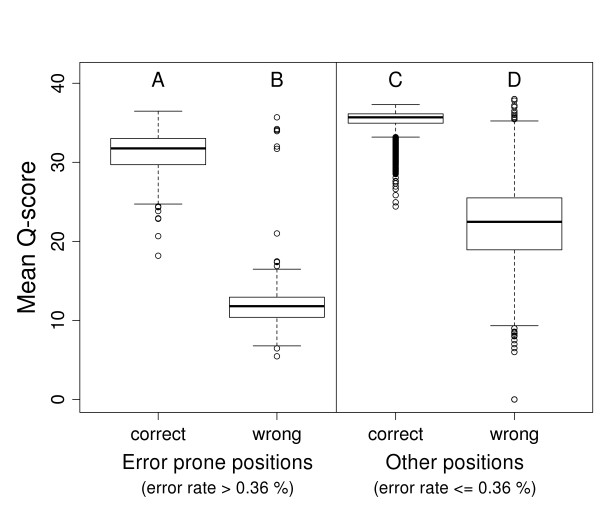
**Mean quality scores of correct and wrong bases sequenced (PhiX-95nt data) at error-prone positions and all other positions in the PhiX reference**. The bases covering 161 positions of significantly elevated error rates (A, B) in the PhiX reference show lower average quality scores compared to the bases covering other positions (C, D). This is true for correctly called bases (A, C) as well as for incorrectly called bases (B, D).

Within the PhiX reference genome we found 161 positions of significantly increased error rates ranging from 0.36% to 8.83% (higher than the mean error rate plus standard deviation). The 161 bases represent 3.0% of the PhiX genome but 24.7% of all substitution errors occur at these positions (PhiX-95nt data). Closer inspection revealed that error rates at these positions differ between the two strands reaching peaks of 18.7% when determining strand-specific error rates (Figure 2d; Table S1 in Additional file 1). We tested the reproducibility by using several other PhiX data sets (generated on both GAIIx and HiSeq instruments; supplemental methods in Additional file 1) and another mapping program. Between different samples and two mapping programs the error prone positions were highly reproducible (Figure S12 and S13 in Additional file 1). However, the finding is less obvious in GA data (Figure S13d-f in Additional file 1) than in HiSeq data (Figure S13a-c in Additional file 1). Among GA data, it is less obvious in the data sets of smaller cycle numbers than in the data set of 150 cycles (Figure S9h in Additional file 1). The 161 positions themselves, but also the surrounding positions, show low average quality values (Figures 2c), and the quality values are low not only for wrongly but also for correctly called bases (Figure 5). The location of these peaks close to regions of accumulated B-tails (Figure 2a) prompted us to trim off a larger part than the actual B-tail (5, 10 and 15 bases more than the B-tail length), but extended trimming and even the complete removal of B-tailed reads could not eliminate the error rate peaks (Figure S14 in Additional file 1). As suggested by B-tail accumulation, we find a non-random distribution of errors within the reference, different for the two strands.

**Figure 6:**
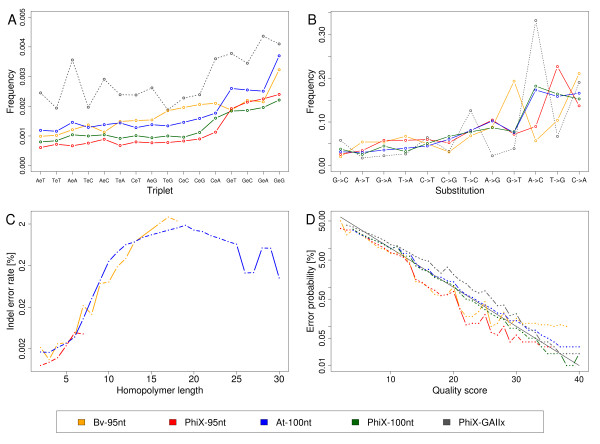
**Frequencies and context of sequencing errors and quality scores compared to observed error rates**. The sugar beet sample (yellow) and the *Arabidopsis *sample (blue) were each sequenced together with PhiX DNA (red and green, respectively) on a HiSeq2000 sequencing instrument. PhiX DNA only (black) was sequenced on a GAIIx. **(a) **Sequence context of substitution errors. The frequency of neighboring bases one position upstream and downstream of an error position is displayed. Sequence triplets were summarized for all types of base substitutions at the central position (indicated by an 'e'). We counted reads spanning the triplet positions and ignored potential further substitution errors within the triplet sequence of the read. The frequency was determined by dividing the occurrence of a triplet containing a central substitution error by the occurrence of all triplets with the same marginal bases but variable central base. The display of triplets is ordered by increasing average frequency in the HiSeq data. **(b) **Frequency of base substitution errors. For each sample, the proportion of each substitution is indicated (ordered by increasing average frequency in the HiSeq samples). **(c) **Rates of insertions or deletions in homopolymer tracts normalized by homopolymer length. Homopolymers longer than seven bases were present only in the two plant samples. Homopolymers of length 16 to 19 in the Bv-95nt data and of length 26 to 29 in the At-100nt data were each covered by less than 50 reads. **(d) **Expected versus observed error rates. Expected error rates according to quality scores (Q) were calculated for Q = 2 to Q = 40 (solid diagonal line). For each sample the uniquely aligned bases were grouped by quality score, and the observed error rate was determined from the number of observed substitution errors for each Q separately.

To determine the probability of particular neighboring bases appearing next to substitution errors, we calculated for all substitution error positions the frequencies of three-base tuples containing the wrong base call at the middle position (Figure 6a). For the bases flanking the wrong base call we used the corresponding reference bases in order to exclude potential additional errors. As a general trend we found substitution errors to be more likely preceded by a G or C than by A or T, which is in agreement with previous reports [2]. In PhiX-95nt the most frequently observed error context G-error-G was 3.9-fold elevated compared to A-error-T. The position after the error is generally more variable than the position before the error, but within tuples starting with the same base the position after the error was more frequently G or A than C or T. When inspecting bases up to five positions preceding an error, G and C were slightly more frequently observed in all templates (Figure S15 in Additional file 1). We paid special attention to the positions of elevated error rates mentioned above and searched for a pattern shared by the sequence context of these positions. Performing a k-mer analysis (K = 3, 4, 5) as well as a simple counting of the four different bases, we inspected the close (5 bp) and distant (200 bp) vicinity upstream and downstream of 136 of the error prone positions (we excluded terminal regions that showed a coverage loss in the linear reference of the circular genome). In the close vicinity we found a high percentage of G (47%) upstream and a slightly higher percentage of A and T (59%) downstream of the error base (average %GC of PhiX = 44.7%). Accordingly, upstream k-mers containing Gs were over-represented, with TGG and AGG showing the highest numbers within the vicinities and GGG and CGG being the most frequent k-mers related to all k-mers of the genome (5-bp as well as 10-bp vicinities tested). In the distant vicinity of 200 bp no significantly over-represented k-mer was found. Nakamura *et al*. [6] reported that GGC was found within the 10-bp vicinities of most of the start positions of error prone regions in their data. We found this motif in the 10-bp vicinities of only 31 of 136 error prone positions in our data. However, start positions of error prone regions detected by Nakamura *et al*. are not necessarily congruent with the single-base positions of elevated error rates we here report (see Discussion).

For a miscalled base three different substitution errors are possible. It was reported previously that in GA I data particular base conversions were more frequently observed than others [2]. We counted all conversion events in our HiSeq and GAIIx data and found again certain preferences. Summarized over all HiSeq data we found A replacing C or vice versa (29.2%) and G replacing T or vice versa (26.8%) to be the most frequent substitutions. The fluorophore groups attached to bases A and C are excited by the same laser and distinguished only by the emission at different wave lengths; the same is true for the fluorophores of bases G and T. The fact that these pairs of bases are exchanged at high frequencies suggests an impact of these detection settings; the emission spectra of bases excited by the same laser might not be perfectly separated.

The individual conversions show slight variation between different HiSeq samples and greater variation between HiSeq and GAIIx samples (Figure 6b). The most frequent conversion in GAIIx data (A into C) is the same as reported for GA I data [2]. In three of four HiSeq samples G was the base most frequently appearing as a miscall (conversion of any other base into G: PhiX-95nt, 38%; PhiX-100nt, 32%; At-100nt, 32%); the Bv-95nt sample had A (33%) as the most frequent resulting base (Table S2 in Additional file 1). The correct base being most frequently called incorrectly was A in At-100nt, PhiX-100nt and PhiX-GAIIx, C in Bv-95nt, and T in PhiX-95nt. We analyzed positions of significantly elevated error rates mentioned above separately. Each of the 136 positions analyzed in the PhiX genome showed a mixture of the three possible substitution errors but in all cases one of them was clearly dominating (seen at fractions of 42.5% to 99.1%). This may lead to confusion with low abundance polymorphisms as observed in heterogeneous samples. Since the individual error rate for the dominating base differed greatly in many cases between the two strands (for 117 of 136 positions by at least 10-fold, for 125 positions by at least 5-fold) a strand-specific analysis can help to distinguish real polymorphisms from region-specific substitution errors by confirming the occurrence of a variation on both strands at about the same rate. Furthermore, as mentioned above, positions of elevated error rates are reflected in the quality values, which should also be taken into account. The conversion from A or T in the reference sequence to G or C in the read sequence was seen at 118 (87%) of the 136 positions as the dominant base substitution and, among these, in 102 cases (86%) the positions were preceded by a G, resulting in G[A/T] being the most frequent motif at positions of elevated error rates. However, this motif occurs many more times (992) in both the forward and reverse strand of the PhiX genome.

### Insertions and deletions

The frequency of insertions and deletions (indels) is very low, and insertions occur less frequently than deletions (Table 5; Table S3 in Additional file 1). The difference in the number of insertions and deletions was larger in the PhiX samples than in the plant samples. Among single-base indels, an insertion or deletion of A or T was more frequently observed than indels of C or G (elevated by an average factor of 7.5 in the plant samples and by a factor of 1.6 in the PhiX samples). Indel events of more than one base occurred at lower rates than single-base indels (14.8% of all indel events in the plant samples, 2.4% in PhiX samples). We calculated the per-base indel error rate in homopolymers of different sizes. Illumina sequencing is considered to be robust against homopolymer errors. However, within homopolymers of increasing lengths from 2 to 15 nucleotides we observed a 1,000-fold increase of the indel error rate per homopolymer base (Figure 6c).

**Table 5:** Number of insertions and deletions

	PhiX-95nt		At-100nt	
	Insertions	Deletions	Insertions	Deletions
All	336 (100%)	3,453 (100%)	11,043 (100%)	15,087 (100%)
1 base of T, A, C or G	330 (98%)	3,381 (98%)	10,225 (93%)	12,936 (86%)
1 base of T or A	259 (77%)	2,100 (61%)	8,878 (80%)	10,940 (73%)
1 base of C or G	71 (21%)	1,281 (37%)	1,347 (12%)	1,996 (13%)
> 1 base	6 (2%)	72 (2%)	818 (7%)	2,151 (14%)

ELANDv2 performs multiseed and gapped alignments, allowing the detection of indels with a length of up to 20 bases. The description of the conditions of ELANDv2 indel calls implies that no indels are reported in terminal regions of the reads. Indeed, simulations showed that no indels were detected if they were located before position 5 or after position 89 within reads of 95 nucleotides. All indels between positions 21 and 76 were reported, and a fraction of the indels was reported for positions 5 to 20 and 77 to 89. Consequently, the indel error rates as displayed in Table 4 can be considered as slightly underestimated.

### Assessment of quality values

Quality scores are relevant for SNP detection and consensus calling and they are also used by mapping programs such as BWA [10] and Bowtie [11]. In all sequenced HiSeq samples the observed error rates fitted well with the expected error rates derived from the quality values assigned by the Illumina base-calling software. The At-100nt and PhiX-100nt data base-called with a newer software version scatter closer around the expected error rates than the Bv-95nt and PhiX-95nt data processed with an earlier version (Figure 6d). Correctly called bases have, on average, a high quality score of 35 to 37 (At-100nt, Q = 37; Bv-95, Q = 36; PhiX-95nt, Q = 35) and wrong called bases have, on average, a low quality score of 18 to 28 (At-100nt, Q = 18; Bv-95nt, Q = 28; PhiX-95nt, Q = 18). We found no major differences when analyzing reads 1 and 2 of the read pair separately (data not shown).

Quality filtering improves the average Illumina quality scores of the sequenced bases at the expense of removing part of the data (see above). We determined expected error rates (calculated from the average quality score) and observed error rates after mapping as well as the fraction of removed bases for different filtering criteria separately and in combination (Table 6; Table S4 in Additional file 1). B-tail trimming reduces observed and expected error rates most drastically. This is a consequence of discarding bases of Q = 2, which is an arbitrary value to mark low quality read segments corresponding to an extremely high error rate of 63%. Uncalled bases were not counted as sequencing errors. If they were counted as sequencing errors, the observed error rates increase slightly by a factor of up to 1.1 (Table S5 in Additional file 1).

**Table 6:** Expected and observed error rates after filtering of aligned reads

	PhiX-Bv			PhiX-GA		
	
	**Expected (%)**^**a**^	**Observed (%)**^**b**^	Percentage bases removed	**Expected (%)**^**a**^	**Observed (%)**^**b**^	Percentage bases removed
No filter	4.549	0.650	0.0	5.829	1.555	0.0
ChF	2.989	0.399	4.8	5.292	1.349	2.0
C33	2.121	0.274	9.3	4.823	1.113	3.3
B-tail	0.166	0.130	7.0	0.194	0.309	9.0
B-tail + ChF	0.137	0.107	9.1	0.182	0.280	9.9
B-tail + N	0.159	0.130	7.2	0.187	0.310	9.5
B-tail + C33	0.106	0.088	12.2	0.172	0.251	10.5
B-tail + A30	0.114	0.092	11.3	0.172	0.262	10.5
B-tail + ChF + C33	0.105	0.087	12.5	0.170	0.248	10.9
B-tail + ChF + A30	0.113	0.091	11.6	0.170	0.257	10.8

^a^Expected error rate: average error probability of each base, assigned by Illumina as Q-scores. ^b^Observed error rate: substitution error rate of aligned bases. ChF, Illumina chastity filter; B-tail, B-tail trimming; N, removal of reads with at least one uncalled base; C33, removal of reads that have less than two-thirds of Q ≥ 30 bases within the first half of the read; A30, removal of reads that have an average Q-score < 30 in the first 30% of the read.

## Discussion

In this work we have analyzed several sequence data sets generated on state of the art Illumina second-generation sequencing instrumentation. Specifically, we analyzed data from the HiSeq2000 and the GAIIx. We determined base substitution and indel error frequencies, and assessed biases of read coverage, sequencing errors, and base quality score assignments.

Based on our analysis of the observed and expected error rates after application of different read filtering steps, we recommend to perform B-tail trimming and to remove reads containing adapter sequence prior to analysis. The accuracy of the sequencing data can be further improved by removing reads that have less than two-thirds of the bases with Q ≥ 30 in the first half of the read, reads not passing the chastity filter, and reads containing at least one uncalled base. However, rigorous quality filtering might reduce the local coverage in regions of accumulated low quality reads. This effect should be taken into account when performing quantitative analyses rather than comparative sequencing. For *de novo *assemblies the coverage loss might result in contig breaks but the accuracy of the consensus sequence will benefit greatly from using error-free input reads; a regionally divergent unfiltered read population will result in either contig breaks or an erroneous consensus.

Despite the improvements of Illumina cluster amplification kits and sequencing reagents, sequencing on the HiSeq and the GAIIx still shows a GC bias. The finding that templates such as ZR with a %GC varying from 24% to 47% (1st and 99th percentiles) show increased coverage of GC-rich regions when using Illumina standard protocols is in accordance with previous results [2,4]. Aird *et al*. [4] analyzed a mixture of genomes covering a broad %GC spectrum and reported a read coverage increase for templates with %GC up to 47% followed by a coverage decrease in regions of higher %GC. Since the %GC of the PhiX is in a narrow interval around the angular point of %GC = 47% (1st and 99th percentiles of PhiX: 41 to 49%) the lack of correlation between read coverage and %GC in PhiX data is in line with the findings of Aird *et al*. The GC bias is also reflected in the %GC of the raw read sequences from the samples we sequenced, which differs from the reference %GC for the two plant species but is close to the %GC of the PhiX genome.

Sequence reads with low base quality can result from, for example, phasing discrepancies. The quality measurement is able to indicate these effects to a certain extent by assigning low quality values of Q = 2, depicted as 'B' in the quality string, typically at the 3' end of reads. Such B-tailed reads were expected to be randomly distributed across the reference genome. However, we observed regions in the reference genome in which the mapping reads have a lower average quality and B-tails accumulate. After removal of B-tails, such regions remained error-free or greatly error-reduced. We found single positions of significantly increased error rates remaining after B-tail trimming. These positions displayed one dominant base conversion, which mainly occurred on one strand.

We were not able to identify sequence-based criteria to predict such error-prone positions unambiguously. Most positions with increased error rates had an over-representation of G in close vicinity upstream and were located within regions of low average base quality values. We found over-representation for several G-containing motifs, especially GGG and CGG. Nakamura *et al*. [6] suggested that a GGC motif precedes error-prone regions. In Nakamura *et al*., the start of such a region is defined by positions of very high error rates (applying the same criteria, we find only one error-prone region in our PhiX-95nt data). In our analysis, we distinguish between two observations: error-prone regions (errors removable by B-tail trimming) and error-prone positions (remaining after B-tail trimming). There does not seem to be any universal short motif that co-occurs with elevated error rates.

We successfully eliminated most error-prone regions by trimming B-tails and retaining the parts of higher quality values. Still, we encountered single positions of elevated error rates, and neither the extension of B-tail trimming towards the 5' end nor complete exclusion of B-tailed reads could remove the error rate peaks at these positions. We speculate that the two phenomena, although related to each other, originate from two effects, one resulting in regions of accumulated errors and low quality, and another being responsible for single positions of drastically increased error rates.

As a single sequence motif could not be found, the co-occurring pattern is expected to be more complex. It has been suggested that folding effects due to inverted repeats might be a reason for the accumulation of errors [6]. We agree that secondary structures might be a potential source of region-specific sequencing artifacts, although the details are not yet understood. The responsible sequence pattern(s) may be located in any part of the fragment to be sequenced, even beyond the actually sequenced end. Pairs of low quality peaks on different strands of the reference should be related to each other, as long as the distance between them does not exceed the library insert size. Closer inspection of low quality regions might reveal the factor(s) causing B-tail accumulation as well as error-prone single positions. For now, we have shown that error-prone regions can be efficiently cleaned by B-tail trimming, and error-prone single positions can be detected by the directionality of the reads and the quality value of the affected base. Low copy single-nucleotide polymorphisms (occurring in viral populations or arising from somatic mutations or RNA editing) have to be distinguished from such sequencing errors. Confirmation on both strands may help to find true variants. Data sets obtained from strand-specific sequencing of RNA are particularly sensitive to such errors as only sequences from one strand are available for analysis. Data interpretation might be complicated in situations when a polymorphic position coincides with an error-prone position and base conversions lead to alterations of allele ratios. Quality values of bases at error-prone positions were found to be clearly reduced, which can serve as an additional criterion for discrimination.

For the Illumina GA I sequencing platform, we previously reported an average error rate of 0.6% for sequencing of ZR with 27-nucleotide reads [2]. For HiSeq data, after B-tail trimming and removal of reads that did not pass the chastity filter, we observed an error rate of 0.16% in reads of 95 bases, testifying to the advanced accuracy of this now matured second generation sequencing technology. In particular, by removing read pairs containing the sequencing adapter, neither HiSeq reads nor the GAIIx reads (data not shown) displayed an exponential increase of error rates towards read ends as reported for GA I data previously. The increase was found to be about two- to three-fold for HiSeq data (95 to 100 nucleotides) and about five- to ten-fold for GAIIx data (150 nucleotides).

For plant DNA sequenced with the HiSeq we obtained slightly higher base substitution rates and indel error rates than for the spiked in PhiX controls. Also, the ratio between insertions and deletions, the ratio between indels of one versus more than one base, and the ratio between A/T versus G/C single base indels were distinctively different in plant and PhiX sequencing data. These differences can potentially be explained by somatic variation present within the plant material from which the DNA was extracted or by the occurrence of consensus errors in the plant reference sequences, for example, within repeat regions, which are difficult to assemble.

## Conclusions

For the successful application of sequencing technologies the read data quality is crucial. We here provided a resource of information regarding several error types as well as ways to detect and minimize bad quality. We showed how appropriate data filtering criteria, inferred from properties of raw reads, substantially reduces error rates. When comparing expected and observed error rates the quality scores assigned by the base-calling software were generally accurate. Within reads, a significantly increased error rate towards the end of the read was not observed after quality filtering. Within the reference sequence, we found regional accumulation of low quality bases and single positions of notably elevated error rates, which are important to consider when analyzing nucleotide variations. Supporting previous recommendations [6,12], we conclude from our data that true variants should be confirmed on both strands and quality values should be taken into account. Error types found in GA data are also present in HiSeq data, such as %GC bias, preferred base conversions, or the presence of a preferred base preceding wrong base calls.

## Materials and methods

### DNA extraction, sequencing library preparation, sequencing

Leaf material from sugar beet (*B. vulgaris*) genotype KWS2320 and from *A. thaliana *accession Columbia (Col-0) were used for DNA extraction. These genotypes were chosen because the same accessions were used in the preparation of the reference genome sequences of the respective species ([13] and unpublished data). Genomic DNA was prepared using the Nucleospin Plant XL Kit (Macherey Nagel, Düren, Germany). The DNA of PhiX174 RF1 was purchased from New England Biolabs (Ipswich, MA, USA). Genomic DNA was fragmented in a Covaris instrument (Woburn, MA, USA) to an average size of 250 nucleotides (plant DNA) or 300 nucleotides (PhiX174 RF1). Library preparation was performed using standard Illumina protocols and Illumina paired-end adapters [14].

Sequencing on the Illumina GAIIx was performed with a paired-end cluster generation kit v4 and TruSeq SBS v5 sequencing kits. A library prepared from PhiX174 RF1 was loaded onto the flowcell at a concentration of 5 pM. Clusters were prepared using the Illumina cluster station according to the manufacturer's instructions. Sequencing was performed following a 2 × 150-nucleotide cycle recipe. For Hiseq sequencing, a PhiX kit v2 library (Illumina) was spiked into the *B. vulgaris *and *A. thaliana *sample libraries at a proportion of about 1% each. The total loading concentration was 7 pM. Amplification was performed in the cBOT (Illumina) using an Illumina HiSeq paired-end cluster generation kit PE-401-1001. For sequencing, a 200 cycle SBS kit FC-401-1001 was used, and 2 × 95 (*B. vulgaris*) or 2 × 100 (*A. thaliana*) cycles of sequencing were performed.

### Data processing, mapping, and read filtering

The Illumina pipeline version 2.8 was used for base-calling of GAIIx data, and the HiSeq2000 control software version 1.1.37 for the *B. vulgaris *sample and version 1.1.37.8 for the *A. thaliana *sample. From the GAIIx run, we obtained a full lane of data consisting exclusively of reads from PhiX. HiSeq data consisted of genomic reads from sugar beet or *Arabidopsis *plus 1% of control PhiX that had been spiked into the genomic sample. HiSeq read pairs were mapped with ELANDv2 (within Casava 1.7) to a PhiX reference sequence provided by Illumina, and consecutively to a sugar beet genome reference sequence prepared and assembled by our group (unpublished data) and sugar beet BAC clone ZR-47B15 insert that had been previously sequenced to finished quality with Sanger dideoxy terminator sequencing chemistry ('ZR', GenBank: FJ752587) [8]. The ZR genotype is the same as the genome reference we prepared (unpublished data). We used this draft genome assembly to select the portion of reads covering ZR. In the first step, we mapped all read pairs of the three sugar beet lanes against the *B. vulgaris *draft assembly. We kept only those pairs of which at least one read mapped to the part of the sugar beet genome corresponding to ZR and nowhere else in the genome. The resulting 37,696 pairs were mapped against the high-quality ZR sequence from Dohm *et al*. [8] and were kept if they had passed the Illumina chastity filter and matched ZR uniquely with the correct read orientation and expected mapping distance of less than 500 nucleotides. Reads passed the chastity filter if they had, within the first 25 cycles, no more than one cycle of a chastity below 0.6 (Chastity = Highest intensity/(Highest intensity + Next highest intensity)). To keep adapter-free pairs only, pairs were removed if the two reads showed the wrong matching order within the reference, that is, if the reverse matching read was found upstream of the forward matching read. This occurs if the read length is larger than the sequenced library insert, resulting in an overlapping read pair containing the Illumina 3' adapter. From the remaining 28,993 pairs we further excluded 4,885 reads that mapped to a region of 6 kbp in ZR at positions 30 to 36 kbp. Within this putatively repetitive region, we found that error rates were elevated five-fold compared to other ZR regions, and the read coverage was five times higher than seen on average in ZR (Figures S1 and S2 in Additional file 1). This region had passed the first filtering step because it was underrepresented in the draft assembly. The final read data set comprises 26,495 read pairs and 111 single reads.

Read match coordinates and information on mismatches were retrieved from the ELAND output file. The Illumina PhiX preparation that was sequenced on the HiSeq contained three base positions that did not correspond to the PhiX reference. Errors at these positions were ignored during analysis.

ELANDv2 performs multiseed and gapped alignment of paired reads. In a mulitseed alignment, in case the first seed (default first 32 bases) cannot be mapped with up to 2 mismatches, the next seed (default next 32 bases) is attempted to be mapped. For *B. vulgaris*/PhiX reads we reduced the seed length to 31 bases to allow up to three seeds in reads of length 95 bp. Starting from the matching seed, the alignment is extended to the full length of the read allowing for more mismatches and gaps (indels) of up to 20 bases. ELANDv2 only opens gaps if a gap corrects at least five mismatches downstream and if the ratio between the number of mismatches in the gapped versus ungapped alignment is above a certain threshold. The latter criterion permits gaps that improve noisy ungapped alignments to be distinguished from *bona fide *small insertions/deletions (CASAVA1.7 User Guide).

### Scripting and data visualization

To create statistics on errors and quality values, we extracted and processed information from the ELAND output using scripts written in Perl v5.8.9 [15] and R 2.9.0 [16]. Plots were generated with R.

### Calculation of per-base indel error rates in homopolymer sequences

Homopolymer sequences in the reference were categorized according to their length. For each of the homopolymer tracts we determined the number of spanning reads and the number of reads with indels. We ignored the first and last ten bases of each read within which indel errors are not reliably called by ELAND. The number of indel errors in homopolymers of a certain size was divided by the number of reads spanning homopolymers of that size. The obtained rates were divided by the homopolymer sizes to obtain the per-base indel error rate in homopolymers (necessary to detect if the indel error rate actually increases with longer homopolymers despite the fact that a longer stretch of sequence can accumulate more indel errors).

### Data availability

Sequence data of this study have been submitted to the Sequence Read Archive (SRP008975).

## Abbreviations

At: *Arabidopsis thaliana*; BAC: bacterial artificial chromosome; bp: base pair; Bv: *Beta vulgaris*; GA: Genome Analyzer; NGS: next generation sequencing; PhiX: bacteriophage PhiX174; SBS: sequencing-by-synthesis.

## Authors' contributions

AEM, JCD, and HH conceived the study. AEM performed data analysis under supervision of JCD. AEM, JCD, and HH interpreted the data and wrote the manuscript.

## Supplementary Material

Additional file 1 **Supplemental text, Figures S1 to S18, Tables S1 to S5, supplemental methods, and supplemental references**. Click here for file
