# Supplementary material for: Evaluation of genomic high-throughput sequencing data generated on Illumina HiSeq and Genome Analyzer systems
Source: Genome Biol. 2011 Nov 8;12(11):R112. doi: 10.1186/gb-2011-12-11-r112 (PMC3334598; doi:10.1186/gb-2011-12-11-r112)
Supplement: Additional file 1 — Supplemental text, Figures S1 to S18, Tables S1 to S5, supplemental methods, and supplemental references. [file gb-2011-12-11-r112-S1.PDF]

# Evaluation of genomic high-throughput sequencing data generated on Illumina HiSeq and Genome Analyzer systems

André E. Minoche[1,2], Juliane C. Dohm[1,2], Heinz Himmelbauer[2*]

[1]Max Planck Institute for molecular Genetics, Ihnestr. 63-73, 14195 Berlin, Germany

[2]Centre for Genomic Regulation (CRG) and UPF, C. Dr. Aiguader 88, 08003 Barcelona, Spain

## Manuscript supplement

## Supplemental text

***T1 Spatial effects on error rates within reads***

A HiSeq flow cell is divided into eight lanes, and each lane is composed of 32 tiles (16 tiles at the top and 16 tiles at the bottom, numbered non-consecutively from 1 to 68) (Figure S11D). We determined the per-cycle error rates for each tile separately and found elevated error rates for single cycles to occur preferentially in tiles 41, 42, 43 and 61 (Figure S16A). These tiles are located at the bottom of the flow cell at one end of the lane. At either end of a lane we find generally higher mean error rates over all cycles, both in HiSeq (Figure S16A, figure S17A) as well as in GAIIx data (Figure S18). These observations suggest that the location of a tile has an impact on the error rate of the reads in that tile both in GAIIx and HiSeq flow cells. However, since the mean quality scores are low for the particular cycles and tiles in HiSeq data (Figure S16B, figure S17B) as well as GAIIx data (not shown) such error positions can be identified without taking a reference sequence into account. Spatial effects on data quality had also been reported for GAIIx 75nt reads by Cox et al. 2010 [1].

## Supplemental figures

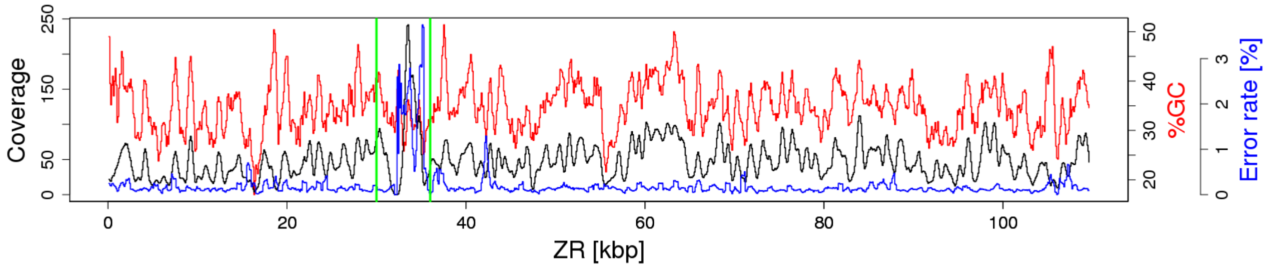


**Figure S1** Read coverage (black), %GC (red) and read error rate (blue) along the sugar beet BAC clone ZR after mapping of Bv-95nt data. Coverage, %GC and error rate were calculated in sliding windows of 500 bases shifted by 100 bases. The region between the green lines (30-36 kbp) has 5-fold elevated coverage and error rates; reads mapping to this region were excluded in further analysis.

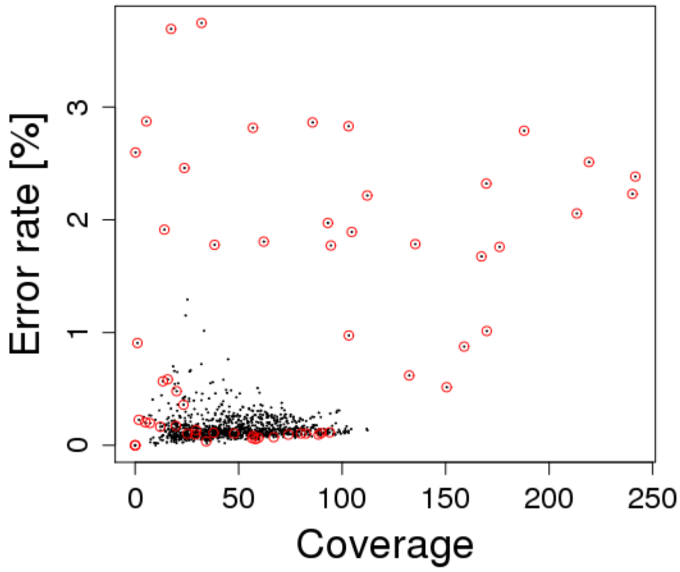


**Figure S2** Read error rate *versus* read coverage of sugar beet BAC clone ZR. Coverage and error rate were calculated in sliding windows of 500 bases shifted by 100 bases. Red circles indicate windows located in the region between 30-36 kbp of ZR which was ignored for further analysis.

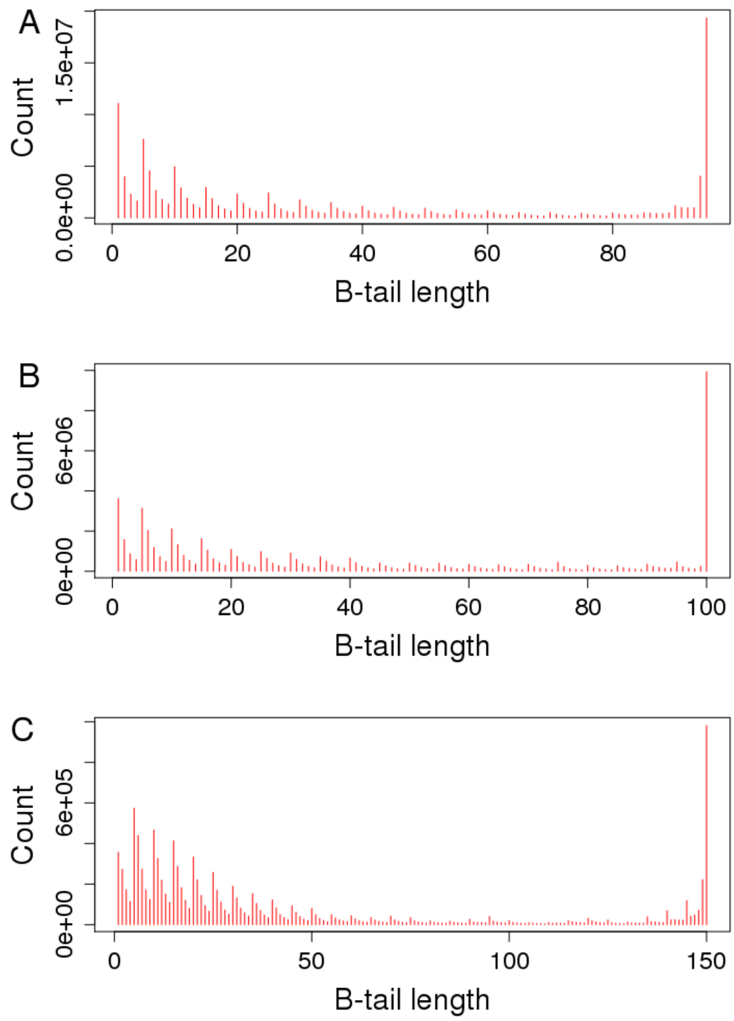


**Figure S3** B-tail length distribution. Reads entirely composed of uncalled bases were not counted. A: Bv-95nt reads; B: PhiX-95nt reads; C: PhiX-GAIIx reads.

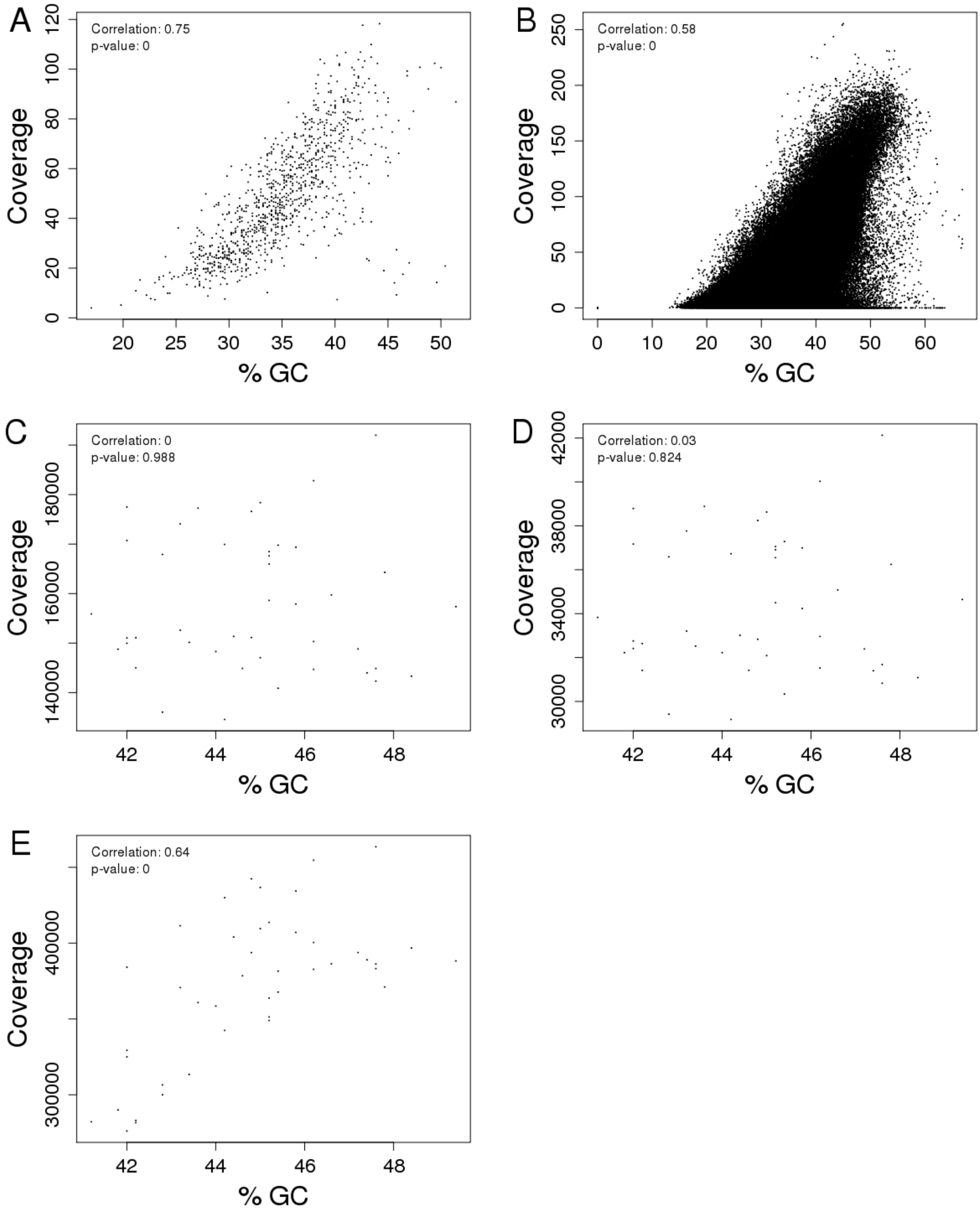


**Figure S4** Read coverage *versus* %GC of the reference sequence for different samples: Bv-95nt (A), At-100nt (B), Phix-95nt (C), Phix-100nt (D), PhiX-GAIIx (E). Coverage and %GC were averaged in sliding windows of 500 bases shifted by 100 bases. The sugar beet sample and the *Arabidopsis* sample were each sequenced together with PhiX DNA on a HiSeq sequencing instrument (Bv-95nt with PhiX-95nt and At-100nt with Phix-100nt, respectively). PhiX DNA only (PhiX-GAIIx) was sequenced on a Genome Analyzer IIx.

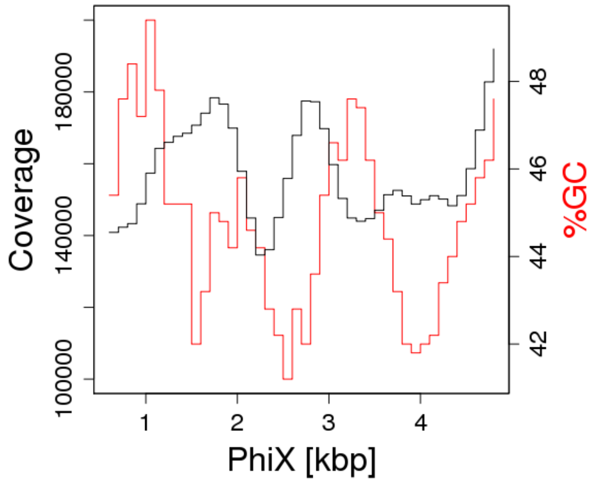


**Figure S5** Coverage (black) and %GC (red) over the PhiX reference (Phix-95nt data). Coverage and %GC were averaged in sliding windows of 500 bases shifted by 100 bases.

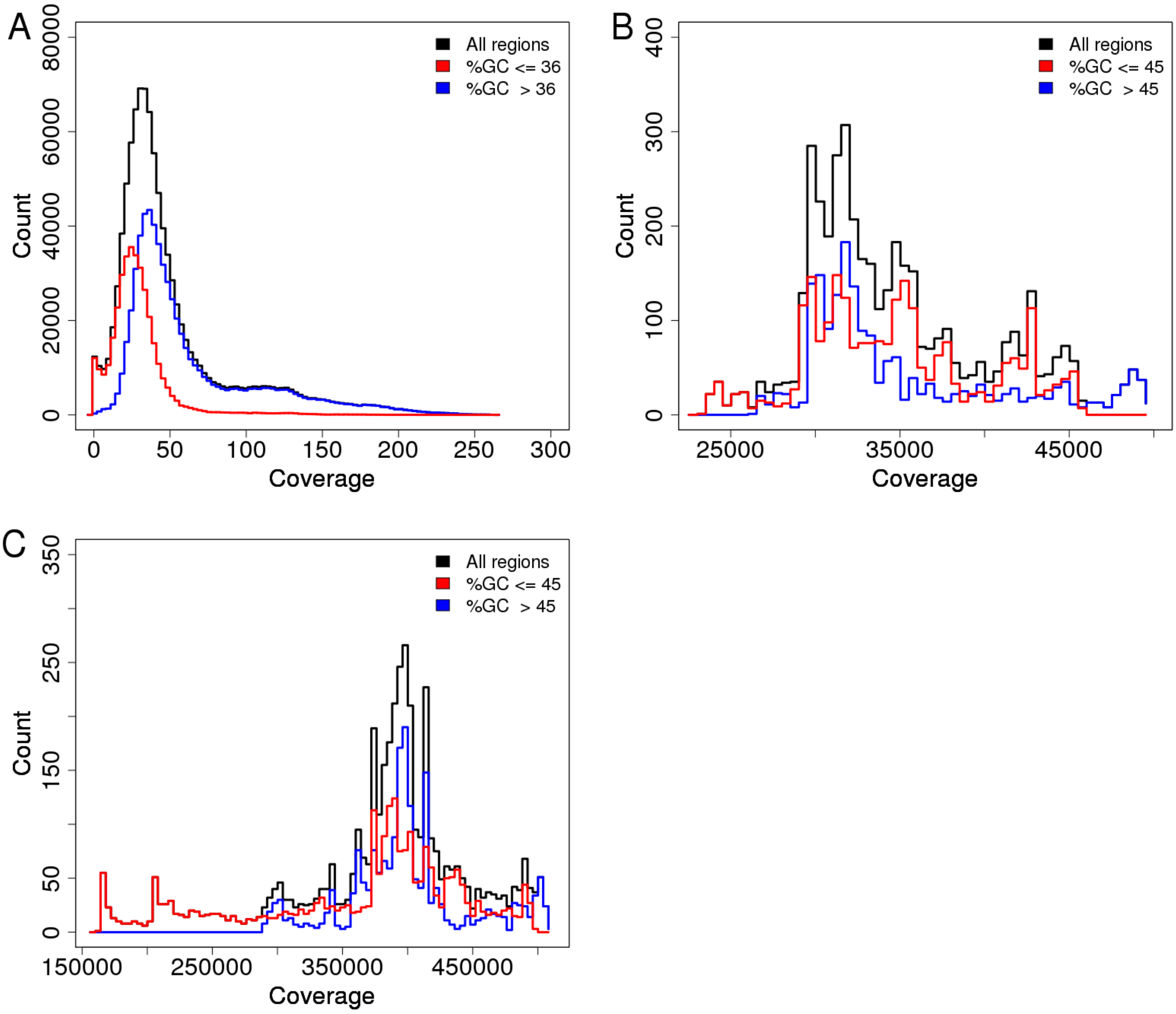


**Figure S6** Distribution of read coverage depth for (A) At-100nt, (B) Phix-100nt and (C) PhiX-GAIIx reads. Read coverage was computed per base. In three separate calculations we considered all positions (black), positions in regions below (red) and positions in regions above (blue) the average GC content of each reference. The regional GC content was determined based on a window of 250 bases upstream and 250 bases downstream of each position.

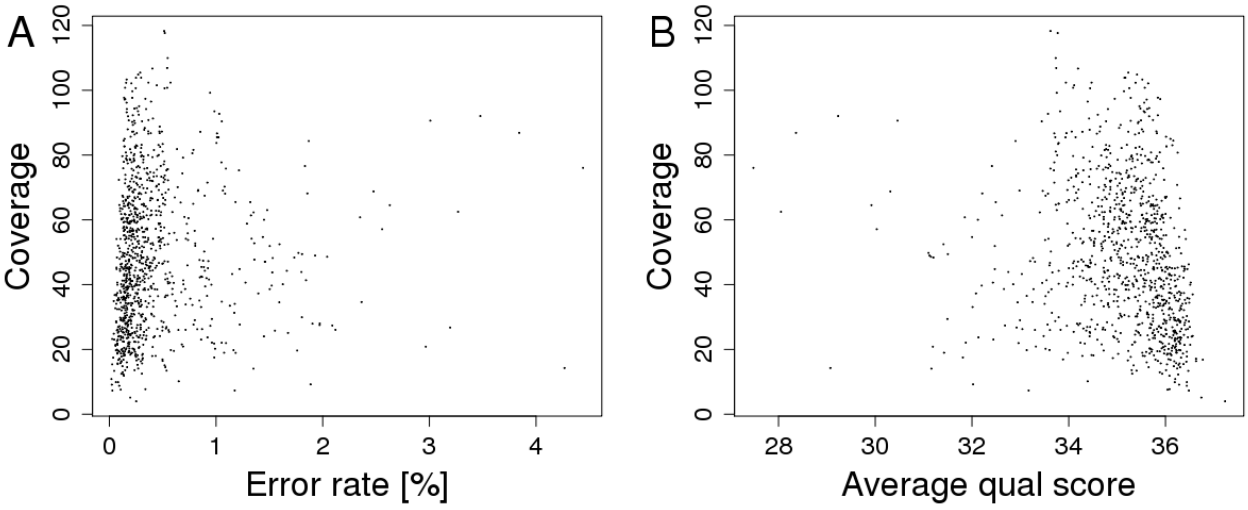


**Figure S7** (A) Average coverage over error rate, (B) average coverage over average quality score of the aligning bases in Bv-95nt data (sliding window of 500 bases, shifted by 100 bases).

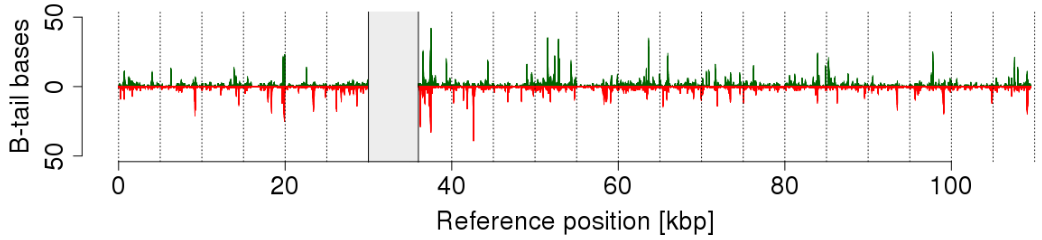


**Figure S8** Location of B-tails along the reference sequence of the BAC clone ZR (Bv-95nt data). Green: forward strand. Red: reverse strand. The interval shown in grey corresponds to a region not included in the analysis (see also Figure S1).

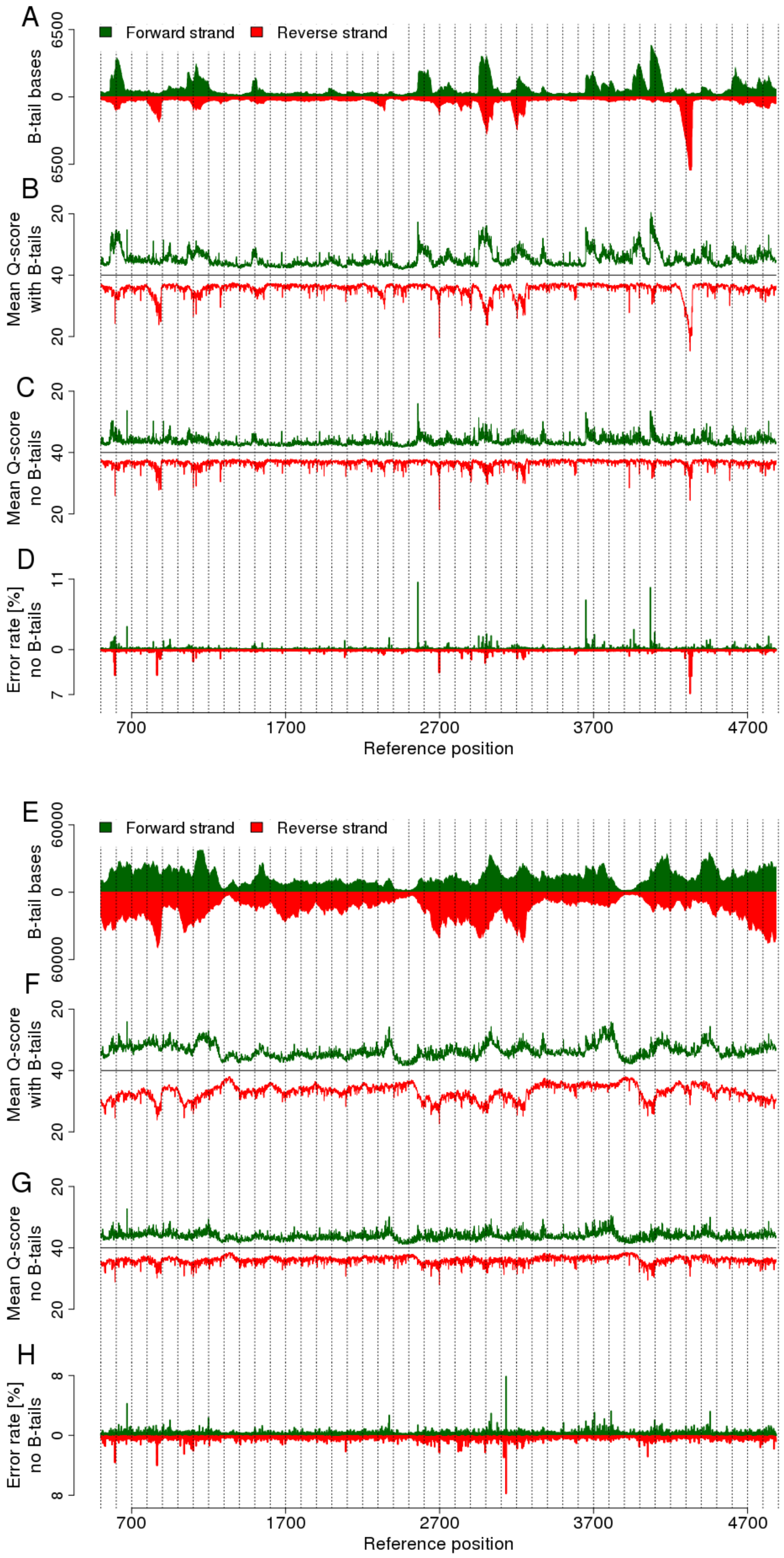


**Figure S9** Distribution of low quality bases along the PhiX reference genome. Analysis was performed on reads derived from two Illumina PhiX libraries. Phix-100nt (A-D) and PhiX-GAIIx (E-H). (A, E) Number of B-tail bases (Q-score=2); (B, F) average Q-score of bases in untrimmed reads; (C, G) average Q-score of bases in B-tail trimmed reads;(D, H) observed per-base substitution error rate. A-H were calculated along the PhiX reference separately for forward (green) and reverse strand (red).

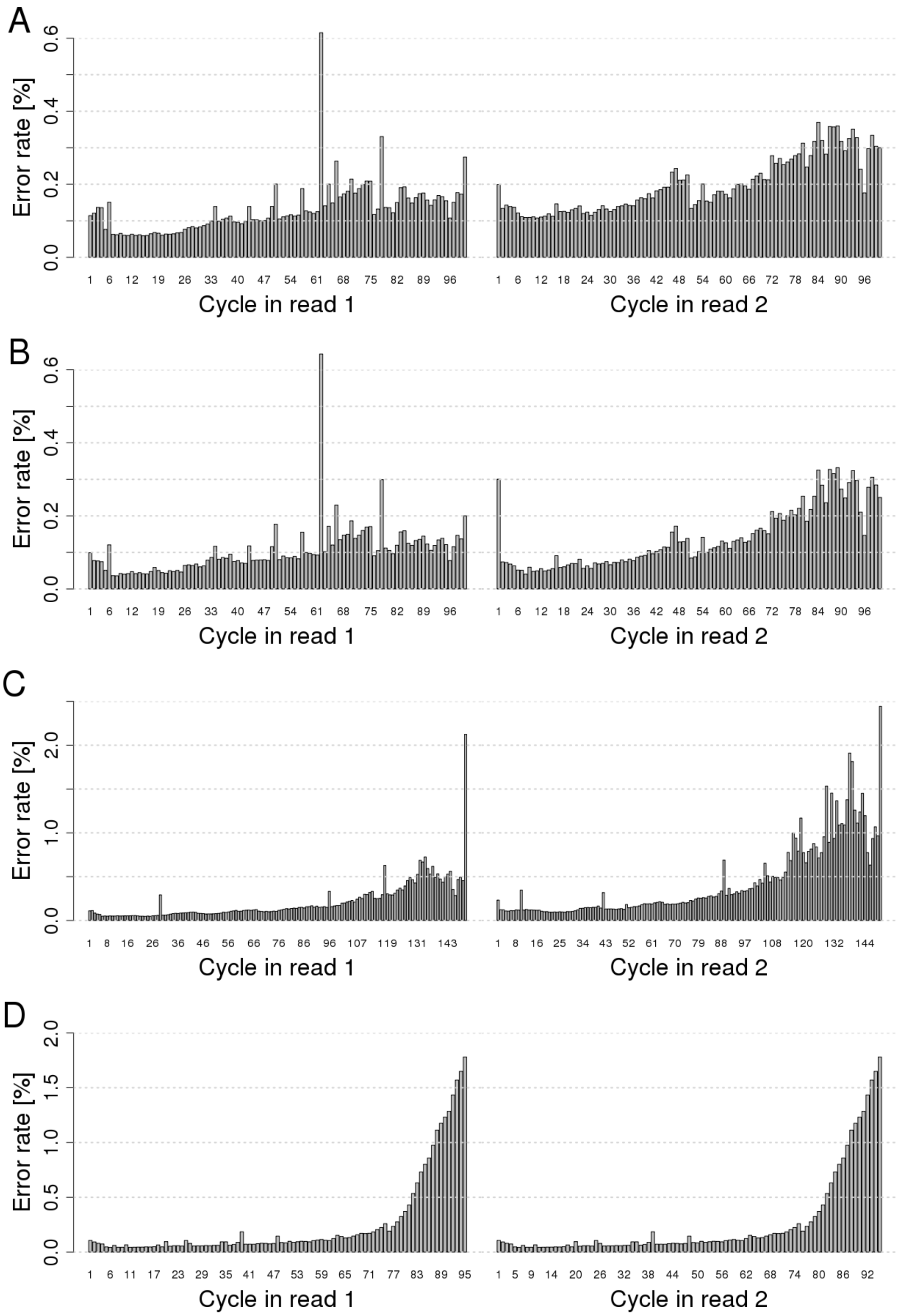


**Figure S10** Observed error rates of the data sets At-100nt (A), Phix-100nt (B), PhiX-GAIIx (C) and PhiX-100nt without adapter trimming (D) by cycle (averaged across all flow cell tiles). At-100nt and Phix-100nt were sequenced together in the same HiSeq lane. Peaks as they appear at cycles 62 and 78 of read 1 in panels A and B result from increased error rates within single tiles (see also Figure S16 A).

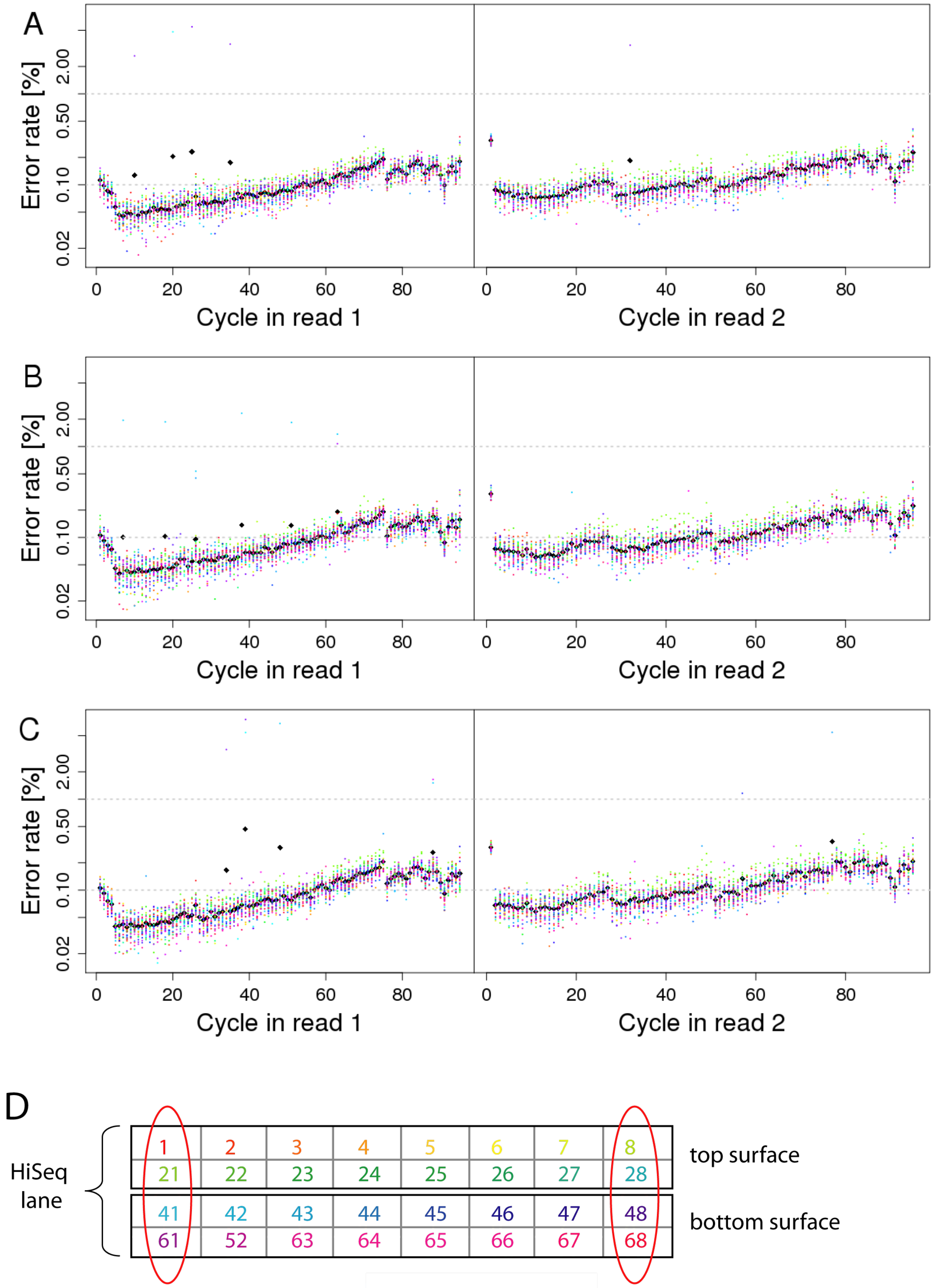


**Figure S11** Per-cycle error rates in the PhiX-95nt data set (PhiX DNA spiked into a genomic sugar beet sample) sequenced in three different HiSeq lanes. The error rates were analyzed separately for each tile of these lanes (A, B, C). Black dots indicate average per-cycle error rates measured across all tiles in one lane. Each tile (in total 32) is plotted in a different color. Increased average error rates occur mainly due to outlier tiles located at lane ends. Panel D shows the spatial arrangement of the tiles of HiSeq flow-cell lanes. Red circles highlight the flow cell ends.

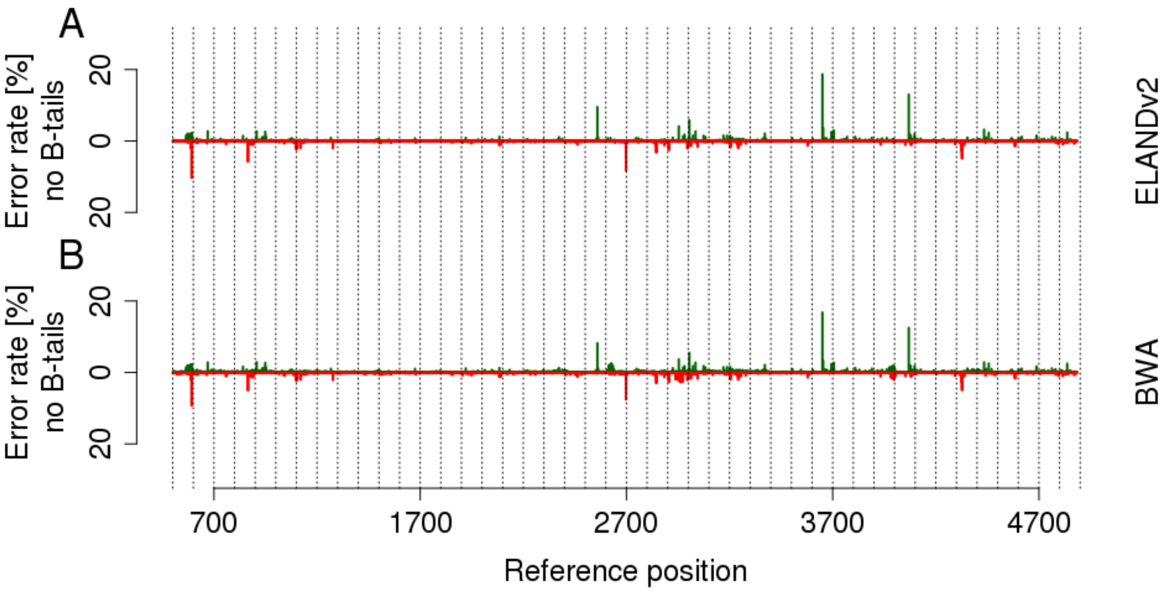


**Figure S12** Comparison of two mapping programs to reproduce positions with increased error rates. B-tail trimmed reads (PhiX-95nt data) were mapped with ELANDv2 (A) and BWA (B). Mapping parameters were adjusted and the same read filtering steps were applied.

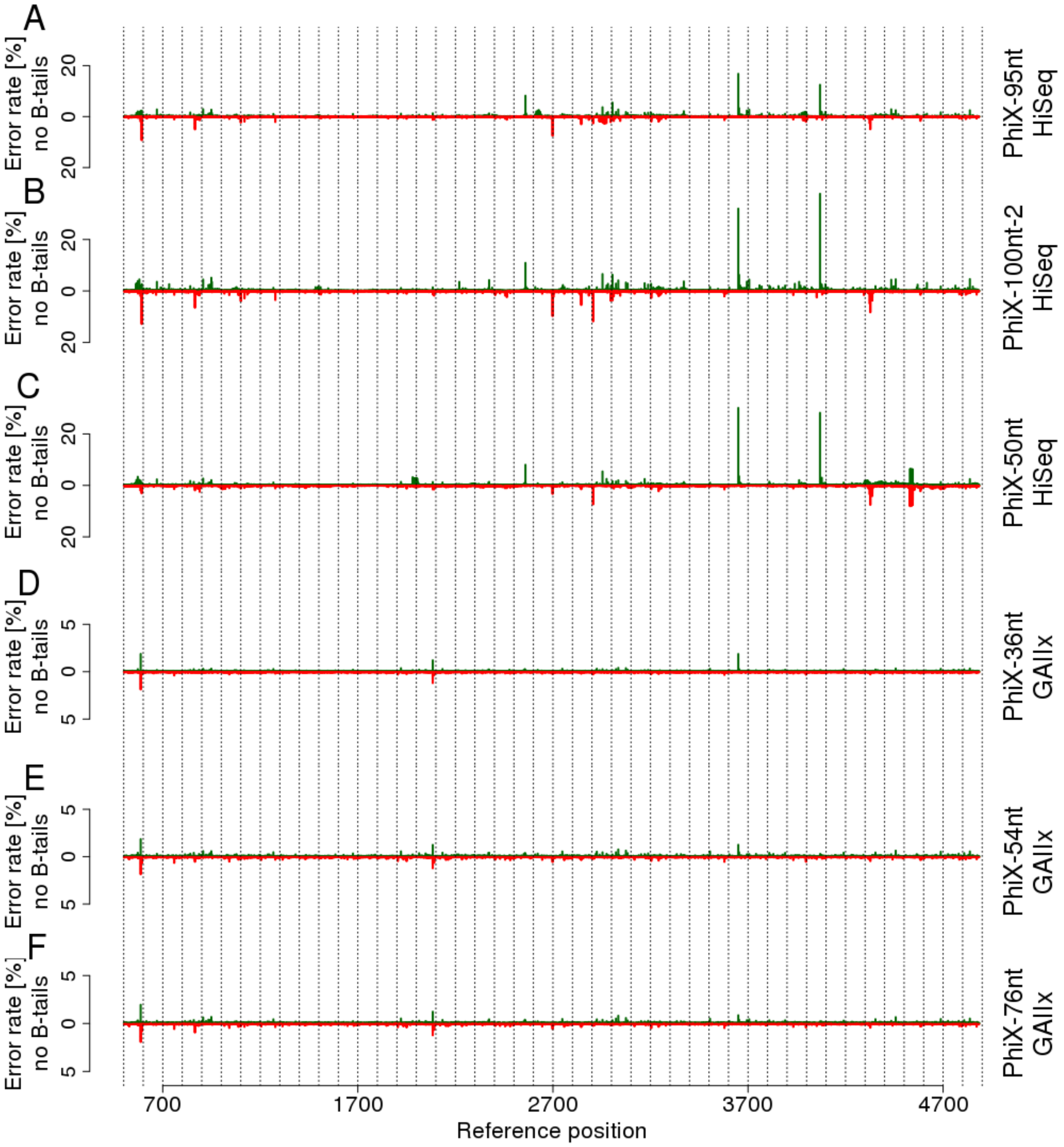


**Figure S13** Comparison of error-prone positions in several PhiX samples sequenced on HiSeq (A-C) and GAIIx (D-F) instruments. Reads were mapped with BWA using a seed size of 31, allowing for 3 edits within the seed and up to 100 edits in total. For PhiX-36nt data the seed size was reduced to 25 bases. Positions with increased error rates are highly reproducible in data generated on the same sequencing platform (see also the result for longer GA reads in Suppl. Fig. S9 H). Error prone positions are much more pronounced in HiSeq samples than in GAIIx samples. For details on sample preparation of the additional data see supplemental methods.

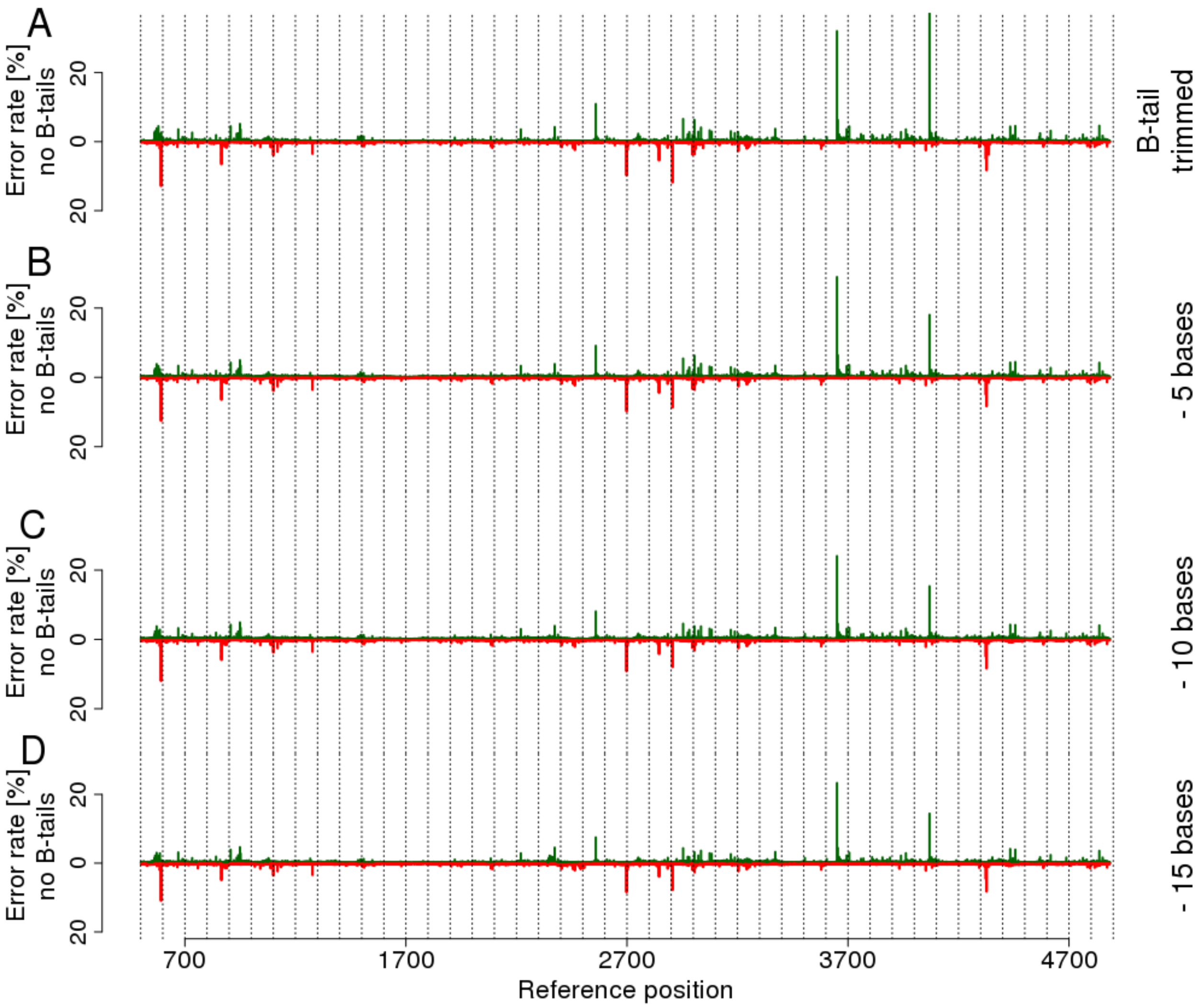


**Figure S14** Effect of extended B-tail trimming on the error rates of error-prone positions. The PhiX-100nt-2 HiSeq dataset was mapped with BWA using B-tail trimmed reads (A) or reads trimmed at 5, 10, or 15 bases upstream of the B-tail start (B-D).

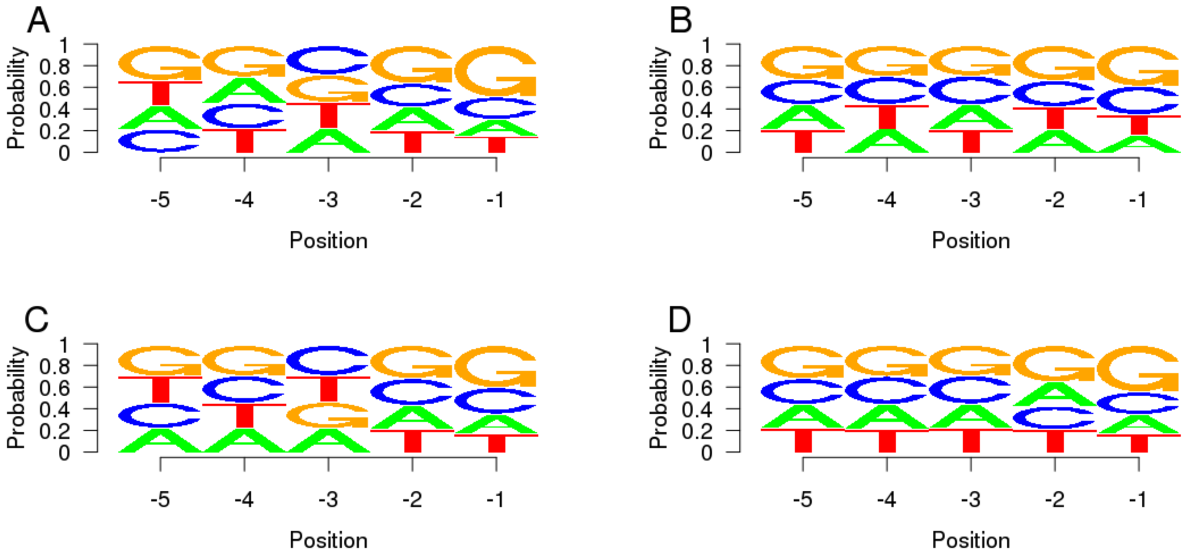


**Figure S15** Base frequencies at positions 5 to 1 upstream of a substitution error. More frequently observed bases are located further above and are indicated by bigger letters (A: Phix-95nt, B: Bv-95nt, C: Phix-100nt, D: At-100nt). The number of individual tuples co-occurring with an error was divided by the overall occurrence of tuples composed of the same sequence motive in reads (wrong base calls within tuples ignored). The sequence logo was created with the seqLogo Bioconductor package in R.

A

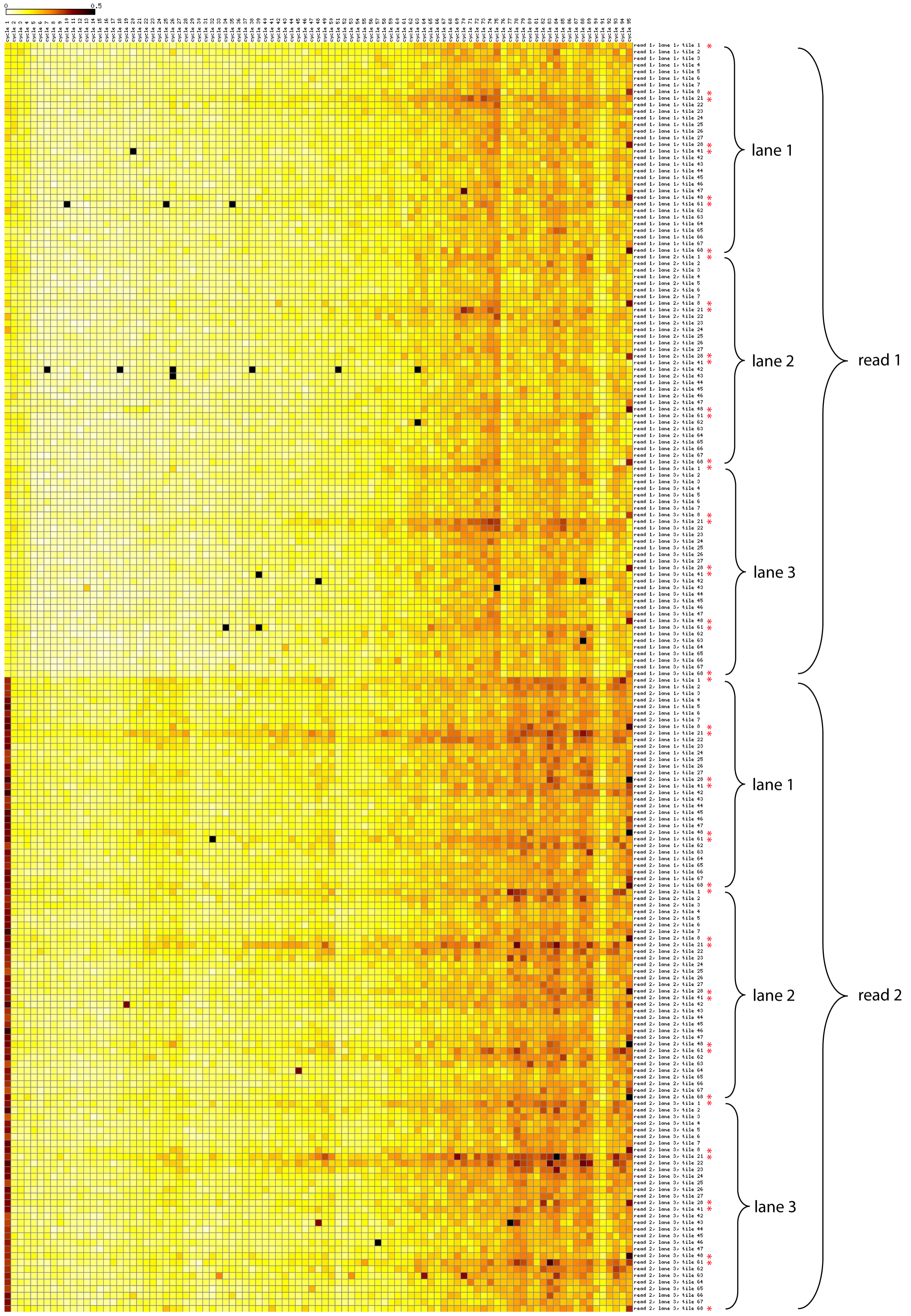

**B**

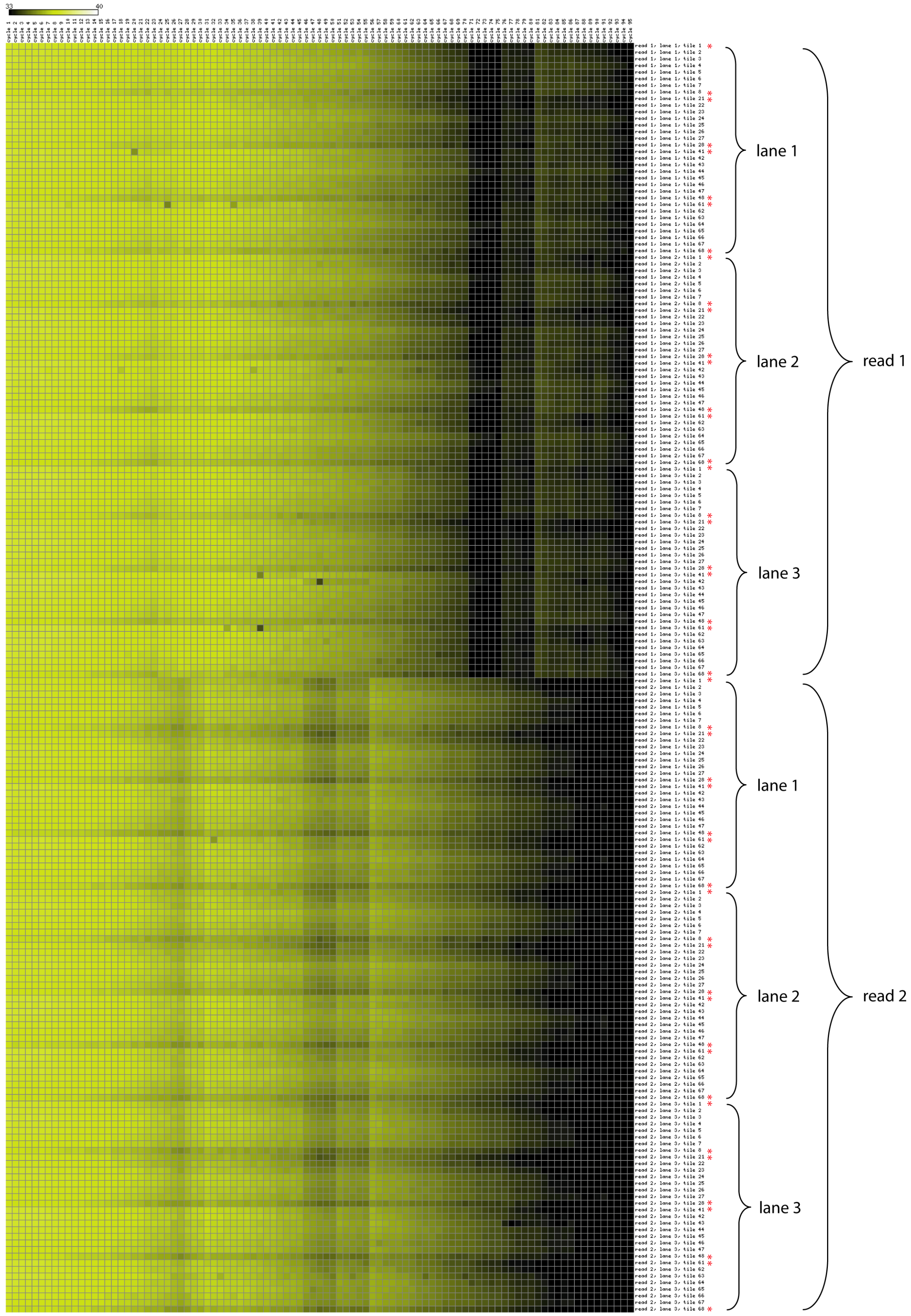


**Figure S16** Heatmaps of per-cycle error rates (A) and per cycle quality values (B) in PhiX-95nt data for each tile and each lane. X-axis: cycle number, y-axis: read names sorted by lane and tile. Heatmaps were generated with Matrix2png [2] similar to Cox et al. [1]. Tiles located at either end of the lane are marked with a red star.

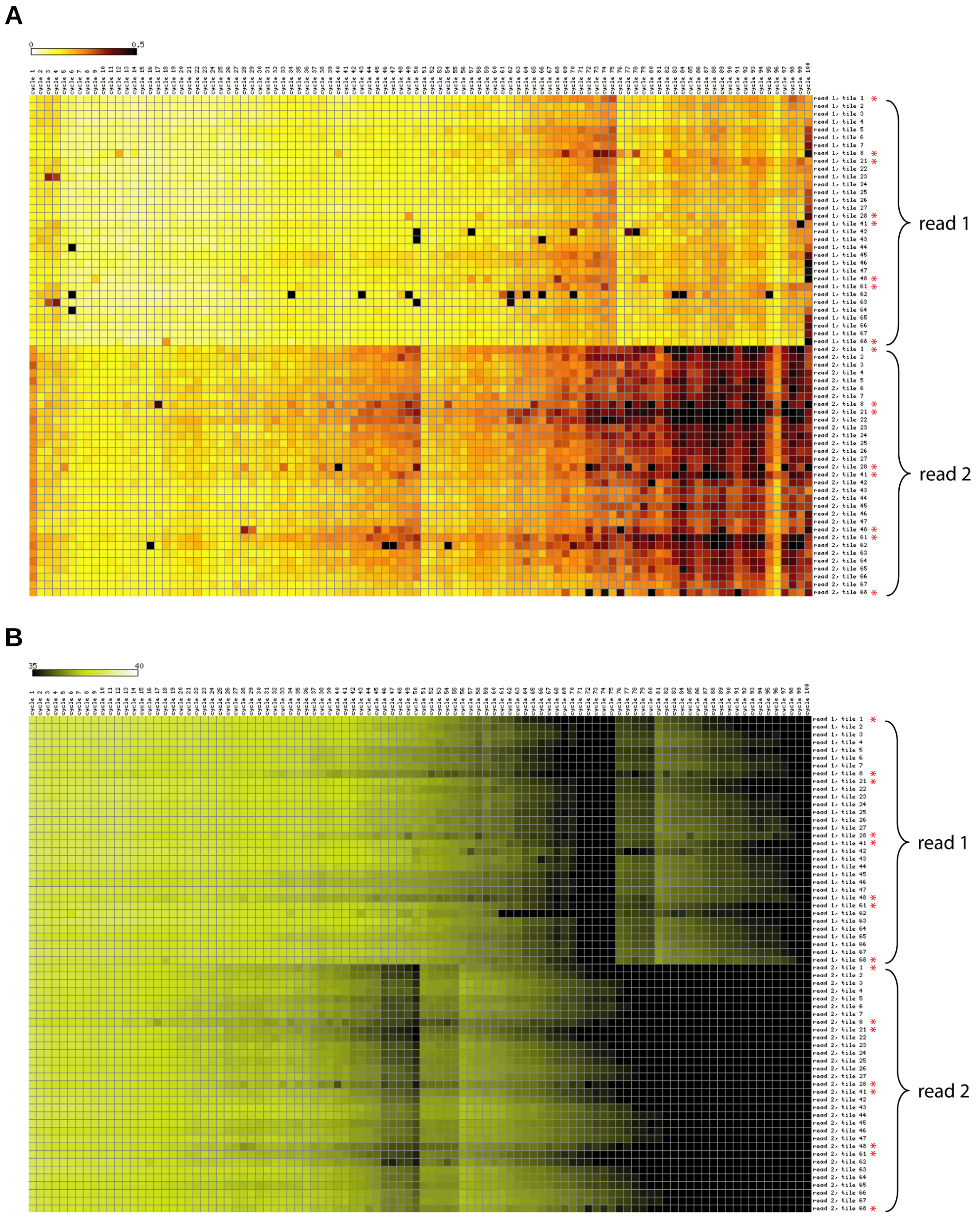


**Figure S17** Heatmaps of per-cycle error rates (A) and per cycle quality values (B) in PhiX-100nt data for each tile and for each lane. X-axis: cycle number, y-axis: read name sorted by lane and tile. Heatmaps were generated with Matrix2png [2] similar to Cox et al. [1]. Tiles located at either end of the lane are marked with a red star.

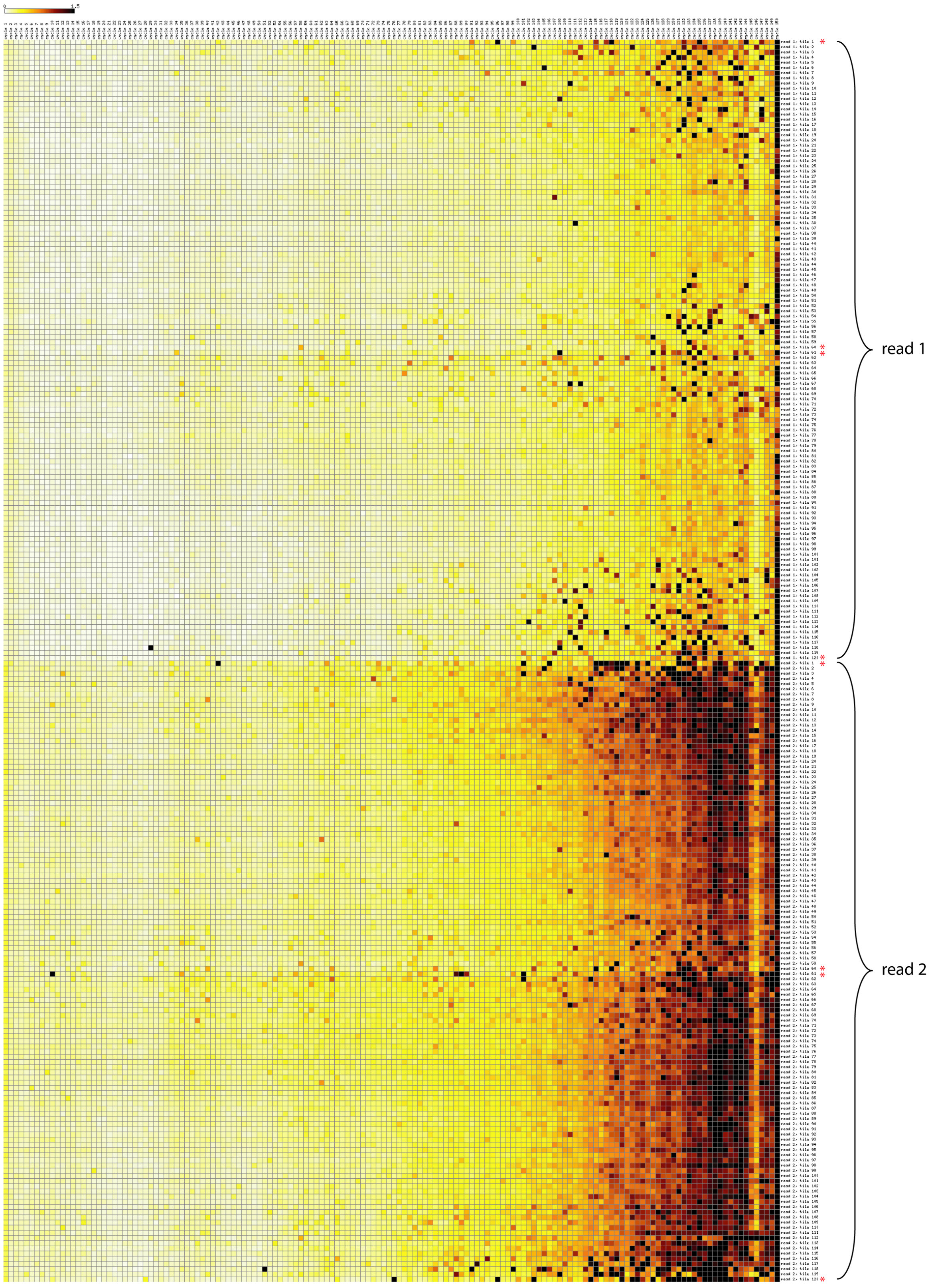


**Figure S18** Heatmap of per-cycle error rates in PhiX-GAIIx data for each tile and lane. X-axis: cycle number, y-axis: read names sorted by lane and tile. The heatmaps were generated with Matrix2png [2] similar to Cox et al. [1]. Tiles located at either end of the lane are marked with a red star.

## Supplemental Tables

**Table S1** List of the 161 positions in the PhiX reference genome with significantly increased error rates (PhiX-95nt reads).

| Position | Error rate [%] Forward | Error rate [%] Reverse | Position | Error rate [%] Forward | Error rate [%] Reverse | Position | Error rate [%] Forward | Error rate [%] Reverse |
|---|---|---|---|---|---|---|---|---|
| 146 | 0.07 | 3.14 | 2884 | 0.04 | 1.23 | 4073 | 1.96 | 0.08 |
| 184 | 0.06 | 1.92 | 2904 | 0.10 | 0.91 | 4074 | 0.75 | 0.07 |
| 565 | 1.56 | 0.04 | 2906 | 0.04 | 2.58 | 4078 | 1.54 | 0.05 |
| 572 | 2.10 | 0.03 | 2925 | 0.95 | 0.05 | 4080 | 0.33 | 1.16 |
| 580 | 1.94 | 0.33 | 2954 | 4.12 | 0.03 | 4097 | 1.97 | 0.04 |
| 587 | 2.11 | 1.89 | 2975 | 1.35 | 0.16 | 4294 | 0.06 | 0.90 |
| 592 | 0.02 | 10.32 | 2978 | 0.94 | 0.11 | 4317 | 0.07 | 0.69 |
| 593 | 2.26 | 0.03 | 2982 | 1.79 | 0.16 | 4324 | 0.04 | 2.74 |
| 617 | 0.05 | 0.60 | 2996 | 0.03 | 2.13 | 4327 | 0.06 | 4.95 |
| 666 | 0.04 | 0.72 | 3005 | 5.84 | 0.24 | 4337 | 0.07 | 1.16 |
| 670 | 2.75 | 0.04 | 3006 | 0.16 | 0.98 | 4353 | 0.97 | 0.08 |
| 733 | 0.67 | 0.04 | 3007 | 0.84 | 0.06 | 4434 | 3.20 | 0.04 |
| 759 | 0.08 | 0.94 | 3012 | 0.29 | 1.45 | 4442 | 0.86 | 0.05 |
| 841 | 1.45 | 0.15 | 3023 | 1.56 | 0.28 | 4446 | 1.00 | 0.05 |
| 859 | 0.63 | 0.16 | 3035 | 2.68 | 0.35 | 4457 | 2.30 | 0.07 |
| 865 | 0.05 | 5.78 | 3040 | 0.36 | 0.38 | 4470 | 0.68 | 0.12 |
| 877 | 0.06 | 0.86 | 3045 | 0.05 | 1.63 | 4565 | 0.67 | 0.03 |
| 883 | 0.07 | 0.80 | 3074 | 1.50 | 0.08 | 4569 | 0.77 | 0.07 |
| 886 | 0.38 | 0.54 | 3083 | 1.14 | 0.03 | 4584 | 0.11 | 1.53 |
| 889 | 0.18 | 1.13 | 3118 | 0.06 | 0.75 | 4600 | 0.63 | 0.11 |
| 892 | 0.08 | 0.66 | 3170 | 1.52 | 0.04 | 4607 | 0.72 | 0.03 |
| 907 | 2.70 | 0.05 | 3176 | 0.63 | 0.48 | 4617 | 1.24 | 0.05 |
| 934 | 1.06 | 0.05 | 3188 | 1.53 | 0.05 | 4688 | 1.54 | 0.07 |
| 941 | 1.14 | 0.03 | 3203 | 1.48 | 0.06 | 4763 | 1.20 | 0.05 |
| 949 | 2.60 | 0.04 | 3204 | 0.65 | 1.83 | 4780 | 0.91 | 0.07 |
| 952 | 1.35 | 0.06 | 3214 | 0.97 | 0.03 | 4783 | 0.35 | 0.65 |
| 1069 | 0.60 | 0.05 | 3232 | 0.66 | 0.20 | 4796 | 0.26 | 0.89 |
| 1078 | 0.78 | 0.07 | 3235 | 0.04 | 0.67 | 4816 | 0.27 | 0.98 |
| 1100 | 0.05 | 2.46 | 3239 | 0.04 | 1.08 | 4837 | 2.37 | 0.06 |
| 1119 | 0.27 | 2.00 | 3242 | 0.62 | 2.03 | 4872 | 0.04 | 0.80 |
| 1134 | 0.18 | 0.59 | 3245 | 0.03 | 1.44 | 4925 | 0.89 | 0.04 |
| 1267 | 0.91 | 0.06 | 3248 | 0.58 | 0.34 | 4967 | 4.20 | 0.06 |
| 1277 | 0.07 | 2.06 | 3255 | 0.56 | 0.94 | 4999 | 11.80 | 0.07 |
| 1494 | 0.55 | 0.10 | 3279 | 0.04 | 0.76 | 5003 | 3.78 | 0.02 |
| 1675 | 0.77 | 0.06 | 3364 | 0.81 | 0.10 | 5034 | 0.92 | 0.07 |
| 1921 | 0.91 | 0.04 | 3371 | 2.11 | 0.04 | 5044 | 0.11 | 0.62 |
| 1950 | 0.09 | 0.71 | 3505 | 0.77 | 0.04 | 5060 | 1.87 | 0.12 |
| 2084 | 1.21 | 1.23 | 3546 | 0.71 | 0.09 | 5094 | 0.06 | 5.45 |
| 2091 | 0.12 | 0.97 | 3579 | 0.05 | 1.30 | 5257 | 0.06 | 0.94 |
| 2258 | 0.29 | 0.45 | 3650 | 18.69 | 0.04 | 5269 | 0.08 | 0.72 |
| 2373 | 1.10 | 0.03 | 3654 | 3.62 | 0.03 | 5275 | 0.05 | 1.18 |
| 2388 | 0.65 | 0.03 | 3663 | 0.83 | 0.05 | 5277 | 0.06 | 0.92 |
| 2464 | 0.04 | 1.03 | 3670 | 0.77 | 0.06 | 5281 | 0.04 | 2.10 |
| 2556 | 0.74 | 0.04 | 3694 | 2.54 | 0.06 | 5284 | 0.06 | 3.98 |
| 2559 | 9.51 | 0.03 | 3706 | 2.96 | 0.03 | 5285 | 2.61 | 0.01 |
| 2563 | 1.00 | 0.03 | 3770 | 1.20 | 0.05 | 5289 | 0.10 | 2.83 |
| 2609 | 0.77 | 0.03 | 3809 | 1.16 | 0.05 | 5290 | 2.66 | 0.02 |
| 2622 | 0.87 | 0.04 | 3890 | 0.93 | 0.03 | 5292 | 0.02 | 3.74 |
| 2645 | 0.27 | 0.54 | 3932 | 0.10 | 1.03 | 5293 | 0.19 | 0.57 |
| 2695 | 0.16 | 0.64 | 3940 | 0.75 | 0.06 | 5295 | 0.13 | 1.34 |
| 2698 | 0.10 | 8.28 | 3962 | 0.88 | 0.05 | 5305 | 6.32 | 0.04 |
| 2748 | 0.91 | 0.05 | 3997 | 0.27 | 0.62 | 5314 | 7.99 | 0.08 |
| 2840 | 0.10 | 0.96 | 4052 | 0.05 | 1.09 | 5378 | 0.00 | 0.50 |
| 2845 | 0.10 | 3.27 | 4069 | 13.01 | 0.10 | | | |

**Table S2** Frequency of substitution errors relative to sum of all substitution errors

**A) PhiX-95nt**

| | From | | | | |
|---|---|---|---|---|---|
| Into | A | C | G | T | Any |
| A | - | 0.14 | 0.06 | 0.06 | 0.25 |
| C | 0.09 | - | 0.03 | 0.08 | 0.20 |
| G | 0.10 | 0.05 | - | 0.23 | 0.38 |
| T | 0.03 | 0.06 | 0.07 | - | 0.17 |
| Any | 0.23 | 0.25 | 0.16 | 0.37 | - |

**B) At-100nt**

| | From | | | | |
|---|---|---|---|---|---|
| Into | A | C | G | T | Any |
| A | - | 0.17 | 0.04 | 0.04 | 0.24 |
| C | 0.17 | - | 0.03 | 0.08 | 0.29 |
| G | 0.01 | 0.06 | - | 0.16 | 0.32 |
| T | 0.03 | 0.05 | 0.08 | - | 0.15 |
| Any | 0.31 | 0.27 | 0.14 | 0.28 | - |

**C) Bv-95nt**

| | From | | | | |
|---|---|---|---|---|---|
| Into | A | C | G | T | Any |
| A | - | 0.21 | 0.05 | 0.07 | 0.33 |
| C | 0.06 | - | 0.02 | 0.07 | 0.15 |
| G | 0.09 | 0.03 | - | 0.10 | 0.22 |
| T | 0.05 | 0.05 | 0.19 | - | 0.30 |
| Any | 0.20 | 0.29 | 0.27 | 0.24 | - |

**D) PhiX-100nt**

| | From | | | | |
|---|---|---|---|---|---|
| Into | A | C | G | T | Any |
| A | - | 0.15 | 0.04 | 0.03 | 0.23 |
| C | 0.18 | - | 0.04 | 0.08 | 0.30 |
| G | 0.09 | 0.07 | - | 0.16 | 0.32 |
| T | 0.03 | 0.05 | 0.08 | - | 0.15 |
| Any | 0.29 | 0.27 | 0.16 | 0.27 | - |

**E) PhiX-GAIIx**

| | From | | | | |
|---|---|---|---|---|---|
| Into | A | C | G | T | Any |
| A | - | 0.19 | 0.02 | 0.03 | 0.24 |
| C | 0.33 | - | 0.06 | 0.13 | 0.52 |
| G | 0.02 | 0.03 | - | 0.07 | 0.12 |
| T | 0.02 | 0.06 | 0.04 | - | 0.12 |
| Any | 0.37 | 0.29 | 0.12 | 0.22 | - |

**Table S3** Number of insertions and deletions

| | PhiX-100nt Insertions | PhiX-100nt Deletions | Bv-95nt Insertions | Bv-95nt Deletions | PhiX-GAIIx Insertions | PhiX-GAIIx Deletions |
|---|---|---|---|---|---|---|
| all | 36 ( 100% ) | 510 ( 100% ) | 25 ( 100% ) | 59 ( 100% ) | 362 ( 100% ) | 6715 ( 100% ) |
| 1 base of T,A,C or G | 36 ( 100% ) | 503 ( 99% ) | 19 ( 76% ) | 51 ( 86% ) | 344 ( 95% ) | 6553 ( 98% ) |
| 1 base of T or A | 19 ( 53% ) | 318 ( 62% ) | 19 ( 76% ) | 44 ( 75% ) | 262 ( 72% ) | 3823 ( 57% ) |
| 1 base of C or G | 17 ( 47% ) | 185 ( 36% ) | 0 ( 0% ) | 7 ( 12% ) | 82 ( 23% ) | 2730 ( 41% ) |
| >1 base | 0 ( 0% ) | 7 ( 1% ) | 6 ( 24% ) | 8 ( 14% ) | 18 ( 5% ) | 162 ( 2% ) |

**Table S4** Expected and observed error rates after filtering of aligned reads

| | At-100nt Expected in % | Observed in % | % bases removed |
|---|---|---|---|
| No filter | 4.217 | 0.938 | 0.0 |
| ChF | 2.938 | 0.706 | 4.0 |
| N | 4.080 | 0.936 | 0.5 |
| C33 | 1.828 | 0.458 | 7.9 |
| **B-tail** | **0.152** | **0.184** | **6.5** |
| B-tail + ChF | 0.131 | 0.161 | 8.3 |
| B-tail + N | 0.147 | 0.184 | 6.7 |
| B-tail + C33 | 0.105 | 0.132 | 10.4 |
| B-tail + A30 | 0.114 | 1.440 | 9.4 |
| B-tail + ChF + C33 | 0.103 | 0.130 | 10.9 |
| B-tail + ChF + A30 | 0.000 | 0.141 | 9.9 |

[1] Expected error rate: average error probability of each base, assigned by Illumina as Q-scores.
[2] Observed error rate: substitution error rate of aligned bases.
**ChF**: Illumina chastity filter; **B-tail**: B-tail trimming; **N**: Removal of reads with at least one uncalled base; **C33**: Removal of reads with less than two-thirds of the bases with Q>=30 in the first half of the read; **A30**: Removal of reads with average Q-score <30 in the first 30% of the read.

**Table S5** Expected and observed error rates after filtering of aligned reads. Uncalled bases counted as errors

| | PhiX-Bv | | | PhiX-GAIIx | | |
|---|---|---|---|---|---|---|
| | Expected[1] in % | Observed [2] in % | % bases removed | Expected [1] in % | Observed [2] in % | % bases removed |
| No filter | 4.549 | 0.721 | 0.0 | 5.829 | 1.589 | 0.0 |
| ChF | 2.989 | 0.444 | 4.8 | 5.292 | 1.380 | 2.0 |
| N | 4.393 | 0.642 | 0.6 | 5.684 | 1.549 | 0.8 |
| C33 | 2.121 | 0.305 | 9.3 | 4.823 | 1.138 | 3.3 |
| B-tail | 0.166 | 0.139 | 7.0 | 0.194 | 0.316 | 9.0 |
| B-tail + ChF | 0.137 | 0.114 | 9.1 | 0.182 | 0.286 | 9.9 |
| B-tail + N | 0.159 | 0.130 | 7.2 | 0.187 | 0.310 | 9.5 |
| B-tail + ChF + C33 | 0.105 | 0.091 | 12.5 | 0.170 | 0.253 | 10.9 |
| B-tail + ChF + A30 | 0.113 | 0.096 | 11.6 | 0.170 | 0.258 | 10.8 |
| B-tail + ChF + C33 + N | 0.103 | 0.087 | 12.7 | 0.167 | 0.249 | 11.2 |
| B-tail + ChF + A30 + N | 0.109 | 0.091 | 11.7 | 0.169 | 0.257 | 10.9 |

**1** Expected error rate: average error probability of each base, assigned by Illumina as Q-scores.
2 Observed error rate: substitution error rate of aligne bases.
**ChF**: Illumina chastity filter; **B-tail**: B-tail trimming; **N**: Removal of reads with at least one uncalled base; **C33**: Removal of reads with less than two-thirds of the bases with Q>=30 in the first half of the read; **A30**: Removal of reads with average Q-score <30 in the first 30% of the read.

## Supplemental Methods

HiSeq sequencing chemistry was as described in Materials and Methods. PhiX-50nt and PhiX-100nt-2 data were basecalled using HCS version 1.1.37.19. PhiX-36nt-GAIIx and PhiX-54nt-GAIIx were sequenced using Illumina v4 flowcells and v5 sequencing chemistry, while PhiX-76nt-GAIIx was prepared using v4 flowcells and v4 chemistry.

The PhiX preparation contains three base positions that do not correspond to the PhiX sequence used as reference. These positions were ignored during analysis.

## Supplemental References

1. Cox MP, Peterson DA, Biggs PJ: **SolexaQA: At-a-glance quality assessment of Illumina second-generation sequencing data**. *BMC Bioinformatics* 2010, **11**:48510.1186/1471-2105-11-485.

2. Pavlidis P, Noble WS: **Matrix2png: a utility for visualizing matrix data**. *Bioinformatics* 2003, **19**:295 -29610.1093/bioinformatics/19.2.295.
